# Supplementary material for: Adjuvant dendritic cell therapy in stage IIIB/C melanoma: the MIND-DC randomized phase III trial
Source: Nat Commun. 2024 Feb 23;15:1632. doi: 10.1038/s41467-024-45358-0 (PMC10891118; doi:10.1038/s41467-024-45358-0)
Supplement: Supplementary file 1 — Supplementary Information [file 41467_2024_45358_MOESM1_ESM.pdf]

Supplementary information

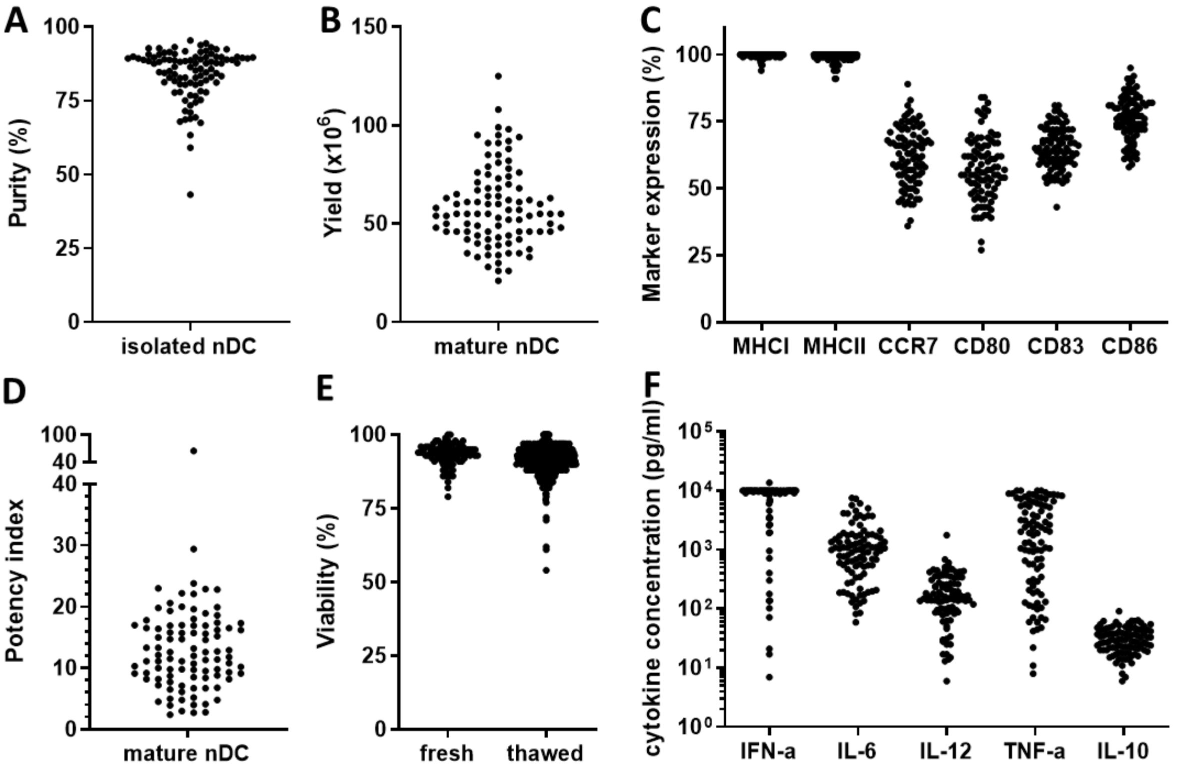

Supplementary Figure 1. Characteristics of freshly isolated and matured natural dendritic cells. (A) Purity of freshly isolated natural dendritic cells (nDCs) was assessed by flow cytometry directly after enrichment with CliniMACS Prodigy. Purity of nDC was defined as the sum of the percentages of CD1c+CD20- cells (cDC2) and CD123+BDCA2+ cells (pDC). The purity of one nDC product was <50% directly after isolation, but 56% in the final product after maturation and thus fulfilled the release criteria of >50% purity. (B) Yield of mature nDCs was determined by cell count of harvested nDC. (C) Phenotype of mature nDCs was assessed by analysis of expression of HLA-ABC (MHC class I), HLA-DR,DP,DQ (MHC class II), CCR7, CD80, CD83, and CD86 by flow cytometry. (D) Potency was analysed in a mixed lymphocyte reaction of mature nDCs and allogenic peripheral blood lymphocytes (PBLs). After 5-6 days of coculturing, T cell activation was analysed by flow cytometry. Potency index is determined as %CD25+ T cells in a coculture of mature nDCs and allogenic PBLs / %CD25+ T cells in a culture of PBLs only. (E) Viability of mature nDCs was determined before cryopreservation (fresh) and after thawing (thawed, n=508). Viability before and after cryopreservation is comparable. (F) Cytokine production was measured by cytometric bead array in the culture medium of mature nDCs after 6 hr of maturation. Each dot represents one nDC product (n=96 in a-d, f). Source data are provided as a Source Data file.

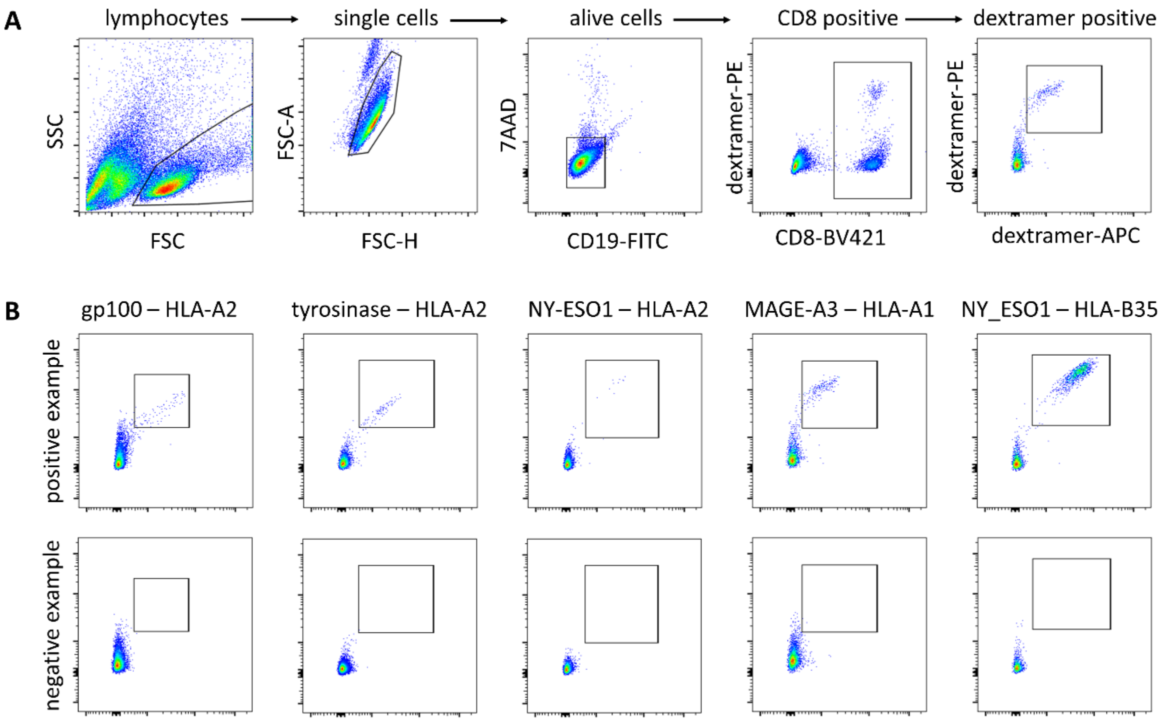

Supplementary Figure 2. Dextramer-analysis of SKILs. (A) Gating strategy. (B) Examples of positive and negative dextramer-staining of SKILs.

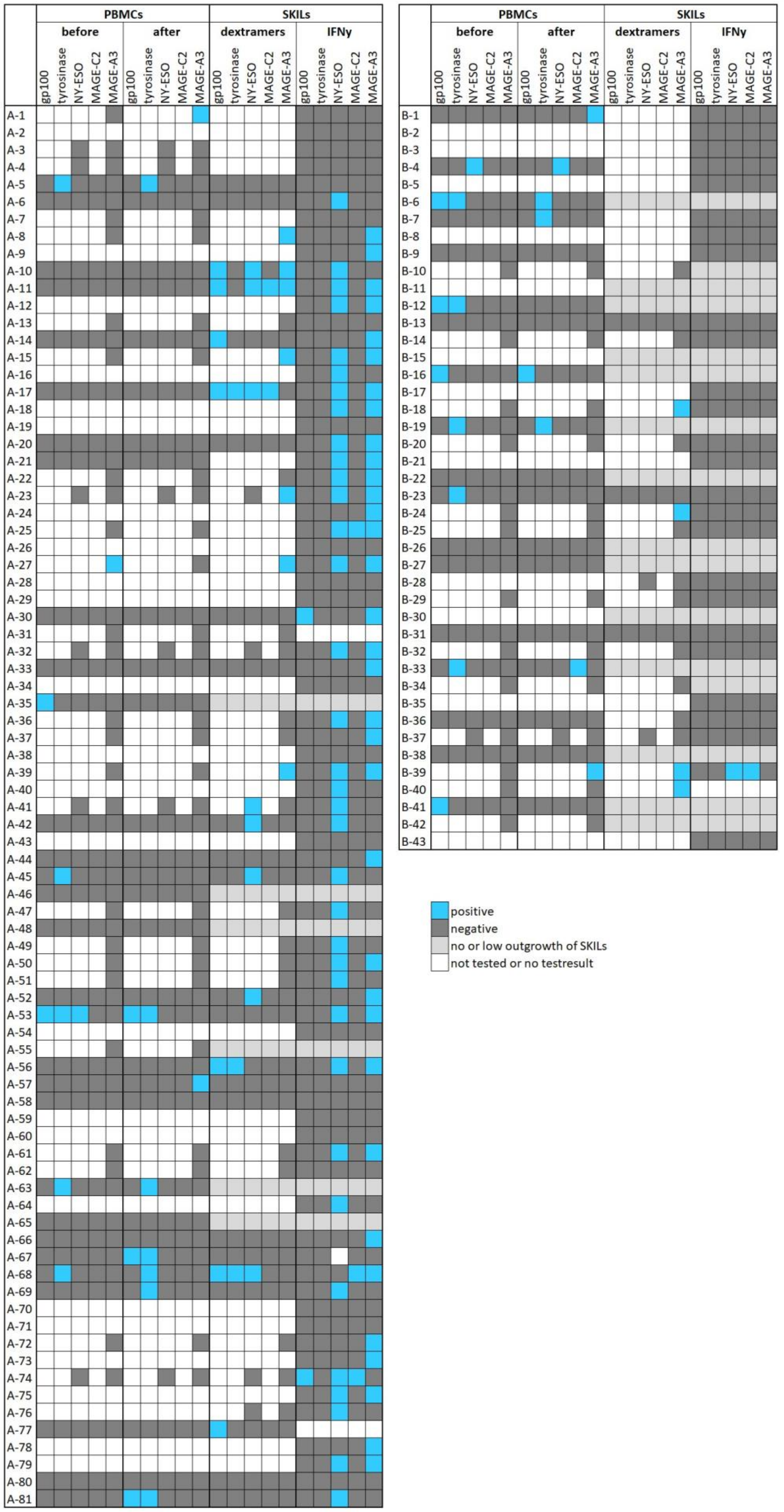

PBMCs
SKILs
before
after
dextramers
IFNγ
gp100
tyrosinase
NY-ESO
MAGE-C2
MAGE-A3
A-1 … A-81
B-1 … B-43
positive
negative
no or low outgrowth of SKILs
not tested or no testresult

Supplementary Figure 3. Antigen-specific T cell responses. Summary of antigen-specific T cell responses per patient and per epitope for patients in the treatment arm (A-1 to A-81) and the placebo arm (B-1 to B-43). Peripheral blood mononuclear cells (PBMCs) were analysed before the first injection and after the first cycle of three injections by dextramer-staining. Presence of antigen-specific skin-test infiltrating lymphocytes (SKILs) was analysed after the first cycle of three injections by dextramer-staining and functionality by analysis of IFNγ production after overnight coculture with antigen-loaded autologous PBMCs. Dextramer-staining of PBMCs or SKILs was only performed on cells of HLA-A2, HLA-A1, or HLA-B35 positive patients. Source data are provided as a Source Data file.

Supplementary Table 1. List of peptides used.

gene	Peptide	Position	HLA	HLA frequency[a]
Gp100	KTWGQYWQV	154-162	A2.1	44%
	YLEPGPVTA	280-288	A2.1	44%
	WNRQLYPEWTEAQRLD	44-59	DR4	24%
Tyrosinase	YMDGTMSQV	369-377	A2.1	44%
	DYSYLQDSDPDSFQD	448-462	DR4	24%
NY-ESO-1	SLLMWITQC	157-165	A2.1	44%
	ASGPGGGAPR	53-62	A3	22%
			A31	6%
	MPFATPMEA	94-102	B35	20%
	PepTivator® NY-ESO-1	Overlapping peptides	Multiple	n.a.
MAGE-C2	ALKDVEERV	336-344	A2.1	44%
	SESIKKKVL	307-315	B44	21%
MAGE-A3	KVAELVHFL	112-120	A2.1	44%
	EVDPIGHLY	168-176	A1	26%
			B35	20%
	EGDCAPEEK	212-220	Cw7	41%
	KKLLTQHFVQENYLEY	243-258	DP4	75%
			DQ6	63%
	PepTivator® MAGE-A3	Overlapping peptides	Multiple	n.a.

Supplementary Table 2. List of antibodies used.

Antibody	supplier	catalog nr	clone	dilution	lot nr	validation information link
anti-CD1c-Viobright-FITC	Miltenyi Biotec	130-113-304	AD5-8E7	1/50	5221203063	CD1c (BDCA-1) Antibody, anti-human
anti-BDCA2-PE	Miltenyi Biotec	130-113-193	AC144	1/200	5230305720	CD303 (BDCA-2) Antibody, anti-human
anti-CD123-APC	Miltenyi Biotec	130-113-322	AC145	1/200	5220906917	CD123 Antibody, anti-human
anti-CD20-PE-Vio770	Miltenyi Biotec	130-113-375	LT20	1/100	5230305724	CD20 Antibody, anti-human
anti-CD45-APC-Vio770	Miltenyi Biotec	130-113-115	5B1	1/800	5220603695	CD45 Antibody, anti-human
anti-CD14-Viogreen	Miltenyi Biotec	130-113-153	TÜK4	1/50	5220908544	CD14 Antibody, anti-human
anti-FcεRI-BioBlue	Miltenyi Biotec	130-123-260	CRA1	1/50	5230305751	CD133/2 Antibody, anti-human
anti-CD14-FITC	Miltenyi Biotec	130-113-146	TÜK4	1/200	5230303439	CD14 Antibody, anti-human
anti-CD15-PE	Miltenyi Biotec	130-113-485	VIMC6	1/100	5230305728	CD15 Antibody, anti-human
anti-CD56-APC	Miltenyi Biotec	130-113-305	AF12-7H3	1/50	5230305719	CD56 Antibody, anti-human
anti-CD3-VioBlue	Miltenyi Biotec	130-113-133	BW264/56	1/50	5220707382	CD3 Antibody, anti-human
anti-HLA-ABC-APC	Miltenyi Biotec	130-120-429	REA230	1/200	5220603710	HLA-ABC Antibody, anti-human, REAfinity™
anti-HLA-DR/DP/DQ-APC	Miltenyi Biotec	130-123-843	REA332	1/400	5220603724	HLA-DR, DP, DQ Antibody, anti-human, REAfinity™
anti-CCR7-APC	Miltenyi Biotec	130-120-600	REA108	1/200	5220908475	CD197 (CCR7) Antibody, anti-human, REAfinity™
anti-CD80-APC	Miltenyi Biotec	130-122-928	2D10	1/200	5220603720	CD80 Antibody, anti-human
anti-CD83-APC	Miltenyi Biotec	130-094-186	HB15	1/160	5221203087	CD83 Antibody, anti-human
anti-CD86-APC	Miltenyi Biotec	130-116-161	REA968	1/300	5220809900	CD86 Antibody, anti-human, REAfinity™ |
anti-CD4-FITC	Biolegend	300506	RPA-T4	1/50	B367819	FITC anti-human CD4 Antibody anti-CD4 - RPA-T4
anti-CD3-PE	BD	555340	HIT3a	1/100	1328405	PE Mouse Anti-Human CD3
anti-CD4-APC	Biolegend	300514	RPA-T4	1/100	B333715	APC anti-human CD4 Antibody anti-CD4 - RPA-T4
anti-CD8-BV421	BD	740093	SK1	1/1000	20180930	BV421 Mouse Anti-Human CD8
anti-CD19-FITC	Miltenyi Biotec	130-113-645	REA675	1/12	5221103028	CD19 Antibody, anti-human, REAfinity™

Supplementary Table 2 continued. List of antibodies used.

Antibody	supplier	catalog nr	clone	dilution	lot nr	validation information link
dextra gp100-154-PE	Immudex	WB2660-PE	A*0201/KTWGQYWQV	5 ul	20170424-IV13	MHC I Dextramer® - Flow Cytometry | Immudex
dextra gp100-154-APC	Immudex	WB2660-APC	A*0201/KTWGQYWQV	5 ul	20170424-IV4	MHC I Dextramer® - Flow Cytometry | Immudex
dextra gp100-280-PE	Immudex	WB3436-PE	HLA-A*0201/YLEPGPVTV	5 ul	201113HM1	MHC I Dextramer® - Flow Cytometry | Immudex
dextra gp100-280-APC	Immudex	WB3436-APC	HLA-A*0201/YLEPGPVTV	5 ul	201113HM2	MHC I Dextramer® - Flow Cytometry | Immudex
dextra tyrosinase-PE	Immudex	WB3245-PE	A*0201/YMDGTMSQV	5 ul	20180116-KB1	MHC I Dextramer® - Flow Cytometry | Immudex
dextra tyrosinase-APC	Immudex	WB3245-APC	A*0201/YMDGTMSQV	5 ul	20180116-KB2	MHC I Dextramer® - Flow Cytometry | Immudex
dextra NY-ESO-1-PE	Immudex	WB3247-PE	A*0201/SLLMWITQV	5 ul	201204HM3	MHC I Dextramer® - Flow Cytometry | Immudex
dextra NY-ESO-1-APC	Immudex	WB3247-APC	A*0201/SLLMWITQV	5 ul	20180116-KB4	MHC I Dextramer® - Flow Cytometry | Immudex
dextra MAGE-C2-PE	Immudex	WB3701-PE	HLA-A*0201/ALKDVEERV	5 ul	20180129-FL46	MHC I Dextramer® - Flow Cytometry | Immudex
dextra MAGE-C2-APC	Immudex	WB3701-APC	HLA-A*0201/ALKDVEERV	5 ul	201204HM7	MHC I Dextramer® - Flow Cytometry | Immudex
dextra MAGE-A3-PE	Immudex	WB3497-PE	A*201/KVAELVHFL	5 ul	20180129_FL48	MHC I Dextramer® - Flow Cytometry | Immudex
dextra MAGE-A3-APC	Immudex	WB3497-APC	A*201/KVAELVHFL	5 ul	20180129_FL49	MHC I Dextramer® - Flow Cytometry | Immudex
dextra HIV-PE	Immudex	WB2194-PE	A*201/SLYNTVATL	5 ul	20170424-IV19	MHC I Dextramer® - Flow Cytometry | Immudex
dextra HIV-APC	Immudex	WB2194-APC	A*201/SLYNTVATL	5 ul	20170424-IV10	MHC I Dextramer® - Flow Cytometry | Immudex
dextra neg control-PE	Immudex	NI3233-PE	-	5 ul	201113HM3	MHC I Dextramer® - Flow Cytometry | Immudex
dextra neg control-APC	Immudex	NI3233-APC	-	5 ul	201113HM4	MHC I Dextramer® - Flow Cytometry | Immudex
dextra NY-ESO-1-PE	Immudex	WK2701-PE	B*3501/MPFATPMEA	5 ul	20180129-FL52	MHC I Dextramer® - Flow Cytometry | Immudex
dextra NY-ESO-1-APC	Immudex	WK2701-APC	B*3501/MPFATPMEA	5 ul	20180129-FL53	MHC I Dextramer® - Flow Cytometry | Immudex
dextra MAGE-A3-PE	Immudex	WK3249-PE	B*3501/EVDPIGHLY	5 ul	20180129-FL56	MHC I Dextramer® - Flow Cytometry | Immudex
dextra MAGE-A3-APC	Immudex	WK3249-APC	B*3501/EVDPIGHLY	5 ul	20180129-FL57	MHC I Dextramer® - Flow Cytometry | Immudex
dextra MAGE-A3-PE	Immudex	WA3249-PE	HLA-A*0101/EVDPIGHLY	5 ul	201204HM4	MHC I Dextramer® - Flow Cytometry | Immudex
dextra MAGE-A3-APC	Immudex	WA3249-APC	HLA-A*0101/EVDPIGHLY	5 ul	201204HM6	MHC I Dextramer® - Flow Cytometry | Immudex

MIND-DC clinical trial protocol

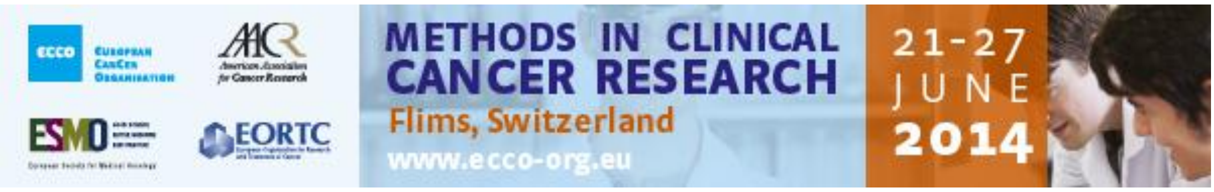

Protocol title: A randomized, double-blind, placebo-controlled phase III study to evaluate active immunization in adjuvant therapy of patients with stage IIIB and IIIC melanoma with natural dendritic cells pulsed with synthetic peptides

Version 4.1 December 2018

Short title: Melanoma patients Immunized with Natural DenDritic Cells (MIND-DC)

Clinical Principal Investigators: Dr. K.F. Bol[1,2]
Prof. dr. W.R. Gerritsen[1]
Prof. dr. J.H.W. de Wilt[3]

Laboratory Principal Investigators: Prof. dr. I.J.M. de Vries[1,2]

[1] Department of Medical Oncology, Radboudumc, Nijmegen, the Netherlands
[2] Department of Tumor Immunology, Radboudumc, Nijmegen, the Netherlands
[3] Department of Surgical Oncology, Radboudumc, Nijmegen, the Netherlands

This protocol was written at the FLIMS workshop 2014.

PROTOCOL TITLE: A randomized, double-blind, placebo-controlled phase III study to evaluate active immunization in adjuvant therapy of patients with stage IIIB and IIIC melanoma with natural dendritic cells pulsed with synthetic peptides.

**Protocol ID**	NL55823.000.15
**Short title**	Melanoma patients Immunized with Natural DenDritic Cells (MIND-DC)
**EudraCT number**	2015-005322-19
**Version**	4.1
**Date**	04 December 2018
**Coordinating Investigator**	Prof. dr. I.J.M. de Vries<br>Department of Tumor Immunology, Radboudumc<br>+31 (0) 24 36 17600<br>Jolanda.deVries@radboudumc.nl
**Principal Investigator(s)**<br><br><br><br><br><br><br><br>**Multicenter research:**	Dr. K.F. Bol<br>Department of Tumor Immunology, Radboudumc<br>Prof. dr. W.R. Gerritsen<br>Department of Medical Oncology, Radboudumc<br>Prof. dr. J.H.W. de Wilt<br>Department of Surgical Oncology<br>Prof. dr. I.J.M. de Vries<br>Department of Tumor Immunology, Radboudumc<br>Radboud university medical centre, Nijmegen<br>Vrije Universiteit medisch centrum, Amsterdam<br>Nederlands Kanker Instituut, Amsterdam<br>Erasmus medisch centrum, Rotterdam<br>Isala klinieken, Zwolle
**Sponsor:**	Radboudumc
**Subsidising party**	Zorginstituut Nederland
**Independent expert (s)**	Dr. N.P.M. Schaap<br>Department of Hematology, Radboudumc

	Michel.Schaap@radboudumc.nl
**Laboratory sites**	Department of Tumor Immunology, Radboudumc, Nijmegen<br>+31 (0) 24 36 17600
**Pharmacy**	Dr. A.L. de Goede – Anna.deGoede@radboudumc.nl<br>+31 (0) 24 36 16405

PROTOCOL SIGNATURE SHEET

Name	Signature	Date
Head of Department:<br>*Prof. dr. C.G. Figdor*<br>Department of Tumor Immunology		
Coordinating Investigator:<br>*Prof. dr. I.J.M. de Vries*<br>Department of Tumor Immunology		
Clinical Principal Investigator:<br>*Dr. K.F. Bol*<br>Department of Tumor Immunology		
Clinical Principal Investigator:<br>*Prof. dr. J.H.W. de Wilt*<br>Department of Surgical Oncology		
Clinical Principal Investigator:<br>*Prof. dr. W.R. Gerritsen*<br>Department of Medical Oncology		

Table of contents

1. Introduction and rationale 14
1.1 Disease background information....14
1.2 Treatment intervention 15
1.2.1 Dendritic cell vaccination 15
1.2.2 Natural dendritic cell subsets 16
1.2.3 Rationale to combine myDC and pDC in cancer immunotherapy....16
1.2.4 nDC maturation with mRNA/protamine as Toll-like receptor ligand 18
1.2.5 Peptide loading of dendritic cells 18
1.2.6 DC-based vaccines in melanoma patients are safe, well tolerated and capable of inducing immunological and clinical responses 19
1.2.7 nDC vaccines in melanoma patients are safe, well tolerated and capable of inducing immunological and clinical responses 20
2. Objectives of the study 25
2.1 Primary objective....25
2.2 Secondary objectives 25
2.3 End-points 25
2.3.1 Primary endpoint 25
2.3.2 Secondary endpoints....25
3. Study population 26
3.1 Number of subjects and patient recruitment 26
3.2 Patient eligibility criteria 27
4. Study design 30
5. Therapeutic regimens, expected toxicity, dose modifications 32
5.1 Investigational treatment 32

5.2 Dose and drug administration 33
5.3 Patient monitoring 33
5.4 Dose adjustments 33
5.5 Duration of therapy 34
5.6 Concomitant therapy 34
6. Clinical evaluation, laboratory tests, follow-up 35
6.1 Study procedures before randomization (screening) 35
6.2 Study procedures during treatment phase (first 1.5 years) 36
6.3 Study procedures during the follow-up phase (last 3.5 years) 37
6.4 Summary tables 38
7. Criteria of evaluation 44
7.1 Primary endpoint 44
7.2 Secondary endpoints 44
7.2.1 Secondary efficacy endpoints 44
7.2.2 Secondary safety and tolerability endpoints 45
7.2.3 Secondary quality of life and cost effectiveness endpoints 45
7.2.4 Immunological endpoints and biomarkers analyses 46
8. Statistical considerations 47
8.1 Statistical design 47
8.1.1 Sample size 47
8.1.2 Randomization and stratification 48
8.2 Statistical analysis 49
8.2.1 Analysis methods for efficacy endpoints 49
8.2.2 Analysis methods for safety, tolerability and cost effectiveness endpoints 50
8.2.3 Analysis methods for immunological endpoints 50

8.3 Interim analysis 50

8.4 Study monitoring 51

9. Quality of life assessment 52

9.1 Rationale 52

9.2 Quality of life instrument 52

9.3 Study design 53

9.4 Statistical considerations 54

10. Economic evaluation 55

10.1 Clinical background 55

10.1.1 Rationale 55

10.1.2 Health economics literature review 56

10.2 Objective 57

10.2.1 Aim of economic study 57

10.2.2 Perspective 57

10.3 Methods 57

10.3.1 Type of economic evaluation 57

10.3.2 Choice of comparator 57

10.3.3 Patient population 57

10.3.4 Measurement of resource use 57

10.3.5 Measurement of unit costs 58

10.3.6 Measurement of effectiveness 58

10.3.7 Time horizon 59

10.4 Statistical analysis 59

10.5 Reporting of results 60

10.6 Collaboration with local health economics research groups 60

11. Patient registration / randomization procedure 61
11.1 Patient identification 61
11.2 Randomization 61
11.3 Method of blinding and breaking the study blind 62
12. Form and procedures for collecting data 63
12.1 Case report forms and schedule for completion 63
12.2 Data flow 63
13. Safety reporting 65
13.1 Section 10 WMO event 65
13.2 Adverse events 65
13.3 Serious adverse events 66
13.4 Suspected unexpected serious adverse reactions 67
13.5 Annual safety report 68
13.5 Follow-up of adverse events 68
14. Quality assurance 69
14.1 Control of data consistency 69
14.2 On-site quality control 69
14.3 Audits 69
15. Ethical considerations 71
15.1 Patient protection 71
15.2 Subject identification 71
15.3 Informed consent 71
16. Administrative responsibilities 73
16.1 Handling and storage of data and documents 73
16.2 Amendments 73

16.3 Annual progress reports 74

16.4 End of study report 74

17. Trial insurance 75

18. Publication policy 76

Appendices 77

Appendix 1. Performance status WHO criteria 77

Appendix 2. Immunomonitoring 78

Appendix 3. Dutch version of the EQ-5D 82

Appendix 4. Dutch version of the EORTC-QLQ-C30 85

Appendix 5. Dutch version of the FACT-M 90

Appendix 6. Dutch version of QoL-MIND-DC questionnaire 96

Appendix 7. Short Health and labour questionnaire 97

List of abbreviations 111

References 114

SUMMARY

Rationale

Melanoma is a highly malignant melanocyte-derived tumor. Standard treatment for patients with stage III disease consists of radical lymph node dissection or observation after limited sentinel-node positive disease. With this treatment patients still have a high risk of local recurrence and development of distant metastases. The life expectancy is therefore limited, with a 5-year overall survival (OS) rate of 67% for stage IIIA, 53% for stage IIIB and 26% for stage IIIC melanoma. No standard adjuvant therapy is currently available that results in a clear clinical benefit. A number of studies carried out throughout the past two decades indicate that melanoma is an immunogenic tumor. The past few years multiple immunotherapeutic approaches have been proven to be successful in metastatic melanoma patients.

We have explored dendritic cell-based immunotherapy. The therapy consists of antigen-loaded autologous DC that are administered to patients with the intention of inducing antigen-specific T and B cell responses. We have now vaccinated well over 300 melanoma patients with monocyte-derived dendritic cell (moDC) vaccines and proved that DC therapy is safe with minimal side effects. Recently we showed that functional anti-melanoma-specific immune responses are observed in about 30% of the metastatic melanoma patients and in about 70% of the stage III melanoma patients, a difference probably due to lower tumor burden in stage III melanoma patients, hence less immunosuppression. Furthermore, we observed a significantly higher overall survival in stage III melanoma patients after adjuvant DC vaccination compared to 209 matched controls, 63.6 months versus 31.0 months ($p=0.018$; hazard ratio 0.59; 95%CI 0.42-0.84).

However, moDC may not be the optimal source of DC for DC vaccination studies, due to extensive culture periods and compounds required to differentiate them into mature moDC. Natural DC (nDC), consisting of plasmacytoid DC and myeloid DC, may be a good alternative since they do not require extensive culture periods. In our previous clinical trials in metastatic melanoma patients using the nDC subsets separately both immunological outcomes, as well as clinical outcomes are promising. Interestingly, the subsets seem to

complement each other functions and act synergistically for optimal immune responses. We therefore hypothesize that co-administration of both subsets may generate more potent and longer-lasting anti-tumor immune responses in cancer patients compared to vaccination with the administration of an individual nDC subset.
After the decades of optimization of DC vaccines it is of importance to re-explore DC vaccination in a randomized fashion in melanoma. Therefore, the aim of this study is to show the potential of nDC vaccination on clinical outcome in high-risk melanoma patients.

Objectives

The primary objective is to determine whether adjuvant treatment with nDC vaccination, after standard treatment in stage IIIB and IIIC melanoma patients, improves recurrence-free survival (RFS) as compared to treatment with matching placebo. The secondary objectives are to compare the median RFS, 2-year and median OS, adverse events profiles (safety), determine immunological responses and factors responsible for the variability of immune responses, quality of life and health economic aspects of nDC vaccination versus placebo.

Study design

This is a phase 3, randomized, double-blind, interventional study of nDC vaccination versus placebo. Subjects will be randomized 2:1 and stratified by stage of disease, adjuvant radiotherapy, BRAF mutation status, HLA-type and nDC production centre. The treatment will be continued for a maximum of 1.5 years or until recurrence of disease, unacceptable toxicity or withdrawal from the study.

Study population

Our study population consists of subjects ≥ 18 years with resection of stage IIIB or IIIC melanoma. Key eligibility criteria are:
- Histologically confirmed, resected stage III cutaneous melanoma, classified as stage IIIB or IIIC disease (AJCC 2009).
- RLND or sentinel node procedure in case of patients without RLND because of limited sentinel-node positive disease must be performed within 12 weeks prior to start of study.
- Absence of distant metastases

- No concomitant use of immunosuppressive drugs orally or intravenously. Topical and intranasal steroids are permitted.
- No uncontrolled infectious disease
- No autoimmune disease. Patients with type 1 diabetes mellitus, hypothyroidism after autoimmune thyroiditis and skin disorders are not excluded.

Intervention

Patients in both arms will undergo a leukapheresis in cycle 1. The patients in the nDC vaccination arm (arm A) will receive 3 nDC injections intranodally ($8x10^6$ nDC) and 4 delayed-type hypersensitivity (DTH) challenges with nDC ($0.5x10^6$ nDC) intradermally injected at the back of the patient. Patients in the placebo arm (arm B) will receive 3 placebo injections intranodally and 4 DTH challenges with placebo intradermally injected at the back of the patient. In both arms punch biopsies will be taken from each DTH site (6mm) and normal skin (3mm) 48 after the DTH challenges. If patients remain clinically free of melanoma, this cycle will be repeated twice with 6-month intervals (without a leukapheresis and DTH test).

Main study endpoints

The primary objective of this study is to determine whether adjuvant nDC vaccination improves 2-year RFS rate as compared to placebo in patients with resected stage IIIB or IIIC melanoma, defined as the percentage of patients who are alive and without recurrence of melanoma 2 years after randomization. Based on previous results on adjuvant DC vaccination in stage IIIB and IIIC melanoma patients, we expect that 70% of the DC vaccinated patients will remain recurrence free for 2 years. The anticipated 2-year RFS rate of the control arm is 50%. The secondary objectives are to compare the median RFS, 2-year and median OS, adverse events profiles (safety), determine immunological responses and factors responsible for the variability of immune responses, quality of life and health economic aspects of nDC vaccination versus placebo.

Burden and risks associated with participation

Based on the experience with the pDC and myDC vaccination separately, we expect that the combined DC vaccine will be well tolerated. Common and expected side effects of DC vaccination are usually mild and include flu-like symptoms and local reaction at injection

site, both not greater than common toxicity criteria grade 1-2. Besides a leukapheresis and DTH test including biopsies in cycle 1 and the 3 intranodal study administrations per cycle (maximum of 3 cycles), upon multiple visits around 100 ml of blood will be drawn for immunomonitoring and safety purposes. Stool, skin- and mouth-swaps will be collected.

1. Introduction and rationale

1.1 Disease background information

Melanoma is staged according to the American Joint Committee on Cancer (AJCC) in 4 stages.[1] Stage III melanoma is defined as melanoma of any thickness with locoregional nodal metastases or in-transit/satellite metastases, in the absence of distant metastases. Stage III melanoma is subdivided according to size and number of nodal metastases and characteristics of the primary tumor (thickness and ulceration). Standard treatment for patients with stage III disease consists of radical lymph node dissection (RLND) or observation after limited sentinel-node positive disease.[2] With this treatment, patients still have a high risk of local recurrence and development of distant metastases. The life expectancy is therefore limited, with a 5-year overall survival (OS) rate of 67% for stage IIIA, 53% for stage IIIB and 26% for stage IIIC melanoma.[3]

Patients with clinically operable positive nodes or in-transit metastasis undergo RLND of the involved site(s) or observation after limited sentinel-node positive disease. No standard adjuvant therapy is currently available that results in a clear clinical benefit. Interferon (IFN)-α is the only adjuvant therapy approved in the USA and EU for the treatment of completely resected stage III melanoma based on significant improvement of recurrence-free survival (RFS) shown in various phase III trials.[4-7] Nonetheless, adjuvant IFN-α is offered only in specialized centers because of substantial toxicity and only minimal OS benefit, at best 3% shown in meta-analysis.[8] In the Netherlands, IFN-α is not administered routinely and there is consensus that adjuvant treatment for stage III melanoma patients should only be administered within the scope of clinical trials with the exception of adjuvant radiotherapy. Recently, ipilimumab (anti-CTLA-4 monoclonal antibodies) was approved by the FDA based on a benefit in RFS and OS.[9, 10]. Ipilimumab has not been approved by the EMA (yet), due to the high toxicity and the changes in the therapeutic landscape of advanced melanoma it is questionable whether ipilimumab will become standard adjuvant treatment in high-risk stage III melanoma. Adjuvant radiotherapy after RLND can be considered for patients with extranodal extension, large metastasis, incomplete surgery or numerous positive lymph nodes to improve regional control, however, without any RFS or OS benefit.[11, 12]

A number of studies carried out throughout the past two decades indicate that melanoma is an immunogenic tumor. It has occasionally been observed that primary tumors as well as metastases disappear spontaneously.[13] In other cases no progression of the tumor is observed for extremely long periods of time (several years). Furthermore, mononuclear infiltrates have frequently been observed and are associated with more favourable prognosis and occasionally tumor regression. Similarly, immune reactivity has been observed in regional lymph nodes. Finally, immuno-suppressed patients, such as transplantation patients, are at higher risk to develop melanoma or other cancer types.[14] These data suggest that the immune system plays an important role in the prevention of melanoma. The role of $CD8^+$ cytotoxic T cells in the eradication of tumor cells that express tumor-associated antigens in the context of Major Histocompatibility Complex (MHC) class I has clearly been established.[15] For melanoma, many tumor epitopes derived from glycoprotein 100 (gp100), tyrosinase, or cancer-testis antigens MAGE and NY-ESO-1 have been identified.

1.2 Treatment intervention

1.2.1 Dendritic cell vaccination

Dendritic cells (DC) are the most potent professional antigen-presenting cells of the immune system and play a central role in immune responses. Upon infection or inflammation, immature DC are activated and differentiate into mature DC that instruct and activate B and T cells, the mediators of adaptive immunity.[16] Currently, DC-based immunotherapy is explored in clinical trials, predominantly in cancer patients.[17, 18] The therapy consists of antigen-loaded autologous DC that are administered to patients with the intention of inducing antigen-specific T and B cell responses. The generation of anti-tumor immune responses involves the induction of T helper 1-type $CD4^+$ T cells and $CD8^+$ cytotoxic T cells.[19-22] Although DC-based immunotherapy frequently induces immunological responses, thus far, a limited number of clinical responses have been observed, but there is evidence that patients developing an immunological response live longer than those without.[23, 24]

To date, monocyte-derived DC (moDC) are used worldwide in clinical vaccination trials. However, it is questioned whether moDC are the most optimal source of DC for the

induction of potent immune responses. The extensive culture period (8-9 days) and compounds required to differentiate them into DC may negatively affect DC migration and function. Especially interleukin (IL)-4 required for the transition of monocytes into immature DC may hamper their capability to migrate.[25, 26] Therefore, it is attractive to consider alternative DC sources, e.g. DC from the blood.

1.2.2 Natural dendritic cell subsets

Two major types of naturally occurring DC (nDC) circulate in the blood,[27] which can be distinguished by the presence of different surface markers. Plasmacytoid DC (pDC) are derived from lymphoid precursors and have plasma-cell like morphology. They produce high amounts of type I IFN in response to viral or bacterial stimuli. Human pDC express CD123, BDCA-2 and BDCA-4, but lack the myeloid antigen CD11c.[28] Myeloid DC (myDC) are characterized by the presence of CD11c and CD1c and have been linked to T helper 1 induction.[29] myDC and pDC express a different repertoire of pattern recognition receptors and respond dissimilar to extracellular stimuli. In addition, the DC subsets exhibit different migration patterns.[30, 31] myDC that reside in almost every organ or tissue generally are considered to exhibit the following in vivo trafficking pattern: Following antigen uptake in peripheral tissues, myDC exit the peripheral tissues in an immature state and traffic via afferent lymphatics to T cell areas of regional lymphoid tissue, where they arrive in an activated state and induce T cell activation and proliferation. By contrast, pDC in the blood follow a different path. During inflammation, immature pDC appear to exit the blood via high endothelial venules for direct entrance into draining lymph nodes.[32] The different expression patterns of pathogen recognition receptors and migratory behavior of pDC and myDC suggest that the DC subsets have functional specializations. The two subsets may therefore act synergistically and contribute both to anti-tumor immunity.

1.2.3 Rationale to combine myDC and pDC in cancer immunotherapy

pDC are very potent inducers of allogeneic T cell responses and prime specific $CD4^+$ and $CD8^+$ lymphocytes against different types of viruses and tumor antigens.[33] In animal models, both myDC and pDC can induce T helper 1 responses. pDC can be found in peritumoral areas and they may affect tumor growth via secretion of soluble factors such as IFN. Interestingly,

pDC and myDC seem to complement each other functions and act synergistically for optimal immune responses. Upon exposure to human immunodeficiency virus (HIV), pDC not only matured and produced cytokines, but also activated bystander myDC, which are not directly activated by HIV.[34] The two DC subsets were also demonstrated to cooperate in antibacterial responses.[35] Indeed using human cells in vitro, we observed that myDC and pDC can activate each other and augment the expression of co-stimulatory molecules (Figure 1). Moreover, in a murine tumor model, immunization with a mix of myDC and pDC proved superior in generating anti-tumor responses when compared to immunization with either DC subset alone.[36] We therefore hypothesize that co-administration of myDC and pDC may generate more potent and longer-lasting anti-tumor immune responses in cancer patients compared to vaccination with the administration of an individual nDC subset.

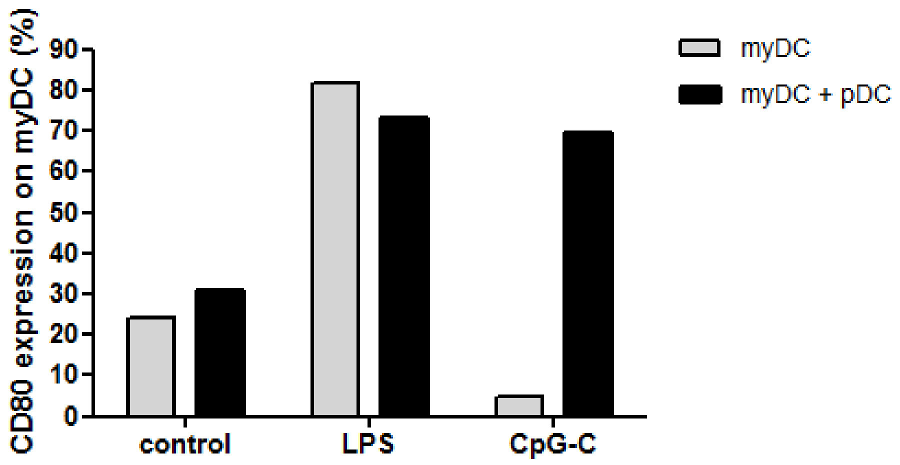

Figure 1. Toll-like receptor-stimulated pDC cross-activate unstimulated myDC. myDC were cultured overnight alone (grey bars) or together with pDC of the same donor (black bars) in the presence of LPS (exclusively stimulates myDC) or CpG-C (exclusively stimulates pDC). After overnight co-culture, CD80 expression, which indicates DC activation, on myDC was analyzed by flow cytometry and. The data show that myDC are not activated by CpG-C when cultured alone, but myDC are activated in the presence of CpG-C when cultured together when pDC, suggesting that CpG-C-activated pDC cross-activate myDC.

1.2.4 nDC maturation with mRNA/protamine as Toll-like receptor ligand

Proper activation of DC is essential prior to re-infusion, particularly because non-activated or incompletely activated DC may cause T cell tolerance rather than productive T cell immunity.[37] Murine studies have shown that activation of DC by solely pro-inflammatory cytokines yield DC that support $CD4^+$ T cell clonal expansion, but fail to efficiently direct helper T cell differentiation. Exposure of moDC to pathogen-associated molecular patterns induces DC that produce high levels of IL-12p70 and promote efficient T cell help.[38, 39] Pathogen-associated molecular patterns are recognized by pattern recognition receptors. Toll-Like Receptors (TLR) are part of this family of proteins and sense microbial and viral products. TLR engagement on DC induces DC maturation and cytokine secretion.[40] TLR ligands enhance survival and expression of costimulatory molecules of both myDC and pDC, and induce IL-12 production from myDC and IFN-α production from pDC, resulting in induction of T helper 1 balance.[41]

Limited TLR ligands are available in Good Manufacturing Practice (GMP) conditions which has impeded the use of TLR ligands for the generation of DC for immunotherapy. We demonstrated that protamine, a GMP grade TLR ligand, in combination with mRNA can induce maturation of both pDC and myDC, yielding clinical grade nDC subsets. Both nDC subsets express high levels of maturation markers and myDC produce T helper 1 polarizing cytokine IL-12p70 whereas pDC produce type I IFN. The characteristics of both subsets stimulated with protamine/mRNA are described in the accompanying Investigational Medicinal Product Dossier (IMPD) “Natural dendritic cell product”.

1.2.5 Peptide loading of dendritic cells

The majority of clinical studies have been performed with MHC class I peptide-pulsed DC. A potential disadvantage of this antigen loading method is that exclusively $CD8^+$ cytotoxic T cells are targeted, without involving $CD4^+$ helper T cells in the induction of anti-tumor responses. It has been shown that the presentation of tumor peptides in both MHC class I and II induces high-affinity T cells reactive to multiple epitopes. Several studies have shown a critical role for helper T cells in the maintenance of long-term protective immunity. Furthermore, helper T cells themselves can also have a direct anti-tumor effect. This effect may be particularly relevant to melanoma, since melanoma cells often constitutively express

MHC class II molecules, which are not down-regulated during progression. In one of our previous clinical trials, we showed that co-targeting both $CD4^+$ helper T cells with moDC pulsed with both MHC class I and II restricted epitopes induced superior vaccine-specific immunological responses and improved clinical responses.[42]
For this trial, we selected melanoma-associated peptides of gp100, tyrosinase, MAGE-A3, MAGE-C2 and NY-ESO-1 because 1) these antigens are widely expressed on primary melanoma and melanoma metastases, 2) derived peptides are able to induce cytotoxic T cells that can lyse tumor cells and 3) the presence of T cells recognizing these peptides in patients suffering from melanoma. We selected peptides binding to molecules frequently observed in the Caucasian population, both Human Leukocyte Antigen (HLA) class I and class II. Due to the wide range of peptides, virtually all patients will be eligible irrespective of their HLA type and antigen expression profile.

1.2.6 DC-based vaccines in melanoma patients are safe, well tolerated and capable of inducing immunological and clinical responses

A number of clinical studies have been performed internationally, including by our own group, in melanoma patients.[43-48] In these studies DC were pulsed with HLA class I-restricted peptides, or with tumor lysate. The conclusions of these studies can be summarized as follows; no severe toxicity has been documented; occasionally an increase of vitiligo or transient constitutional symptoms has been recorded. The studies differed in dose, schedule and route of DC administration, as well as in the use of peptides, adjuvant, and control antigens
Objective clinical responses have been documented, however in a minority of patients. Functional anti-melanoma-specific immune responses are observed in about 30% of the stage IV melanoma patients (n=69) [23, 24] and in about 70% of the stage III melanoma patients (n=78).[49] A correlation between the presence of a tumor-specific immune responses after DC vaccination and OS was noted. Furthermore, OS was significantly higher after adjuvant DC vaccination compared to 209 matched controls, 63.6 months versus 31.0 months (p=0.018; hazard ratio 0.59; 95%CI 0.42-0.84; Figure 2).

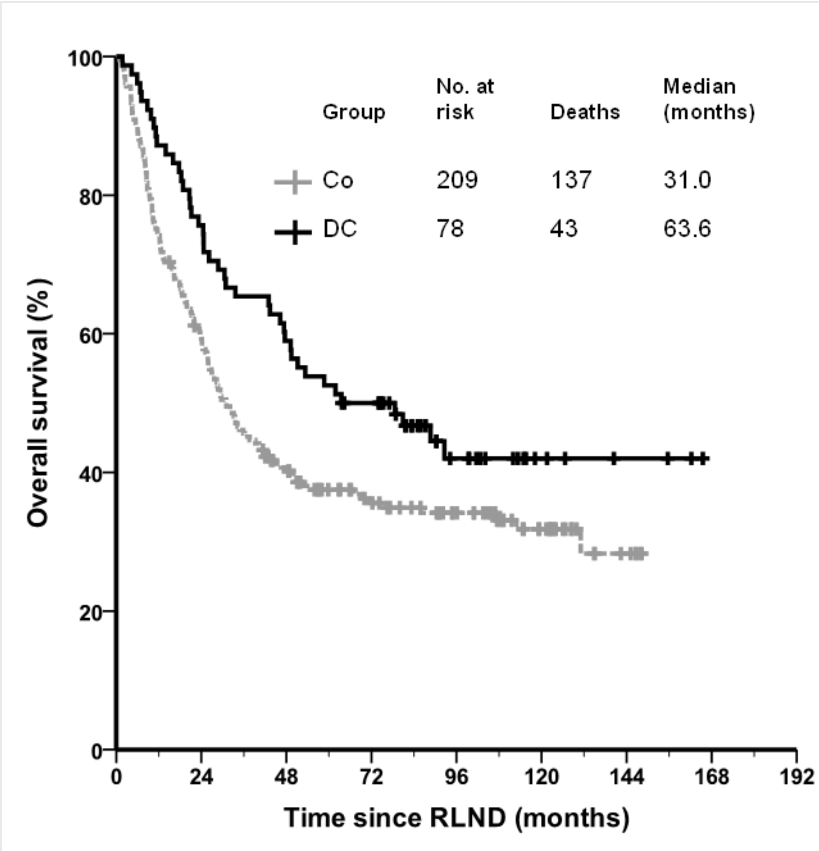

Figure 2. DC vaccination improves overall survival in stage III melanoma patients. Kaplan-Meier curve of patients with regional metastasized melanoma who received adjuvant dendritic cell vaccination after radical lymph node dissection (DC) or no adjuvant treatment (Co).

1.2.7 nDC vaccines in melanoma patients are safe, well tolerated and capable of inducing immunological and clinical responses

We performed a clinical trial in stage IV melanoma patients using pDC.[50] The results on both immunological outcome, especially type I IFN production, as well as clinical outcome are promising. Although the initial endpoint of that study was safety and feasibility, the median OS of pDC vaccinated patients (n=15) showed remarkable improvement compared to control patients (n=72) who received dacarbazine chemotherapy as first-line treatment, the standard treatment at that time, matched on M substage, number of metastases and baseline serum lactate dehydrogenase (LDH) (Figure 3). In patients receiving moDC vaccinations, we did not observe such a suggestive increase in OS, suggesting that pDC vaccines may induce even more potent anti-tumor responses than moDC vaccines.

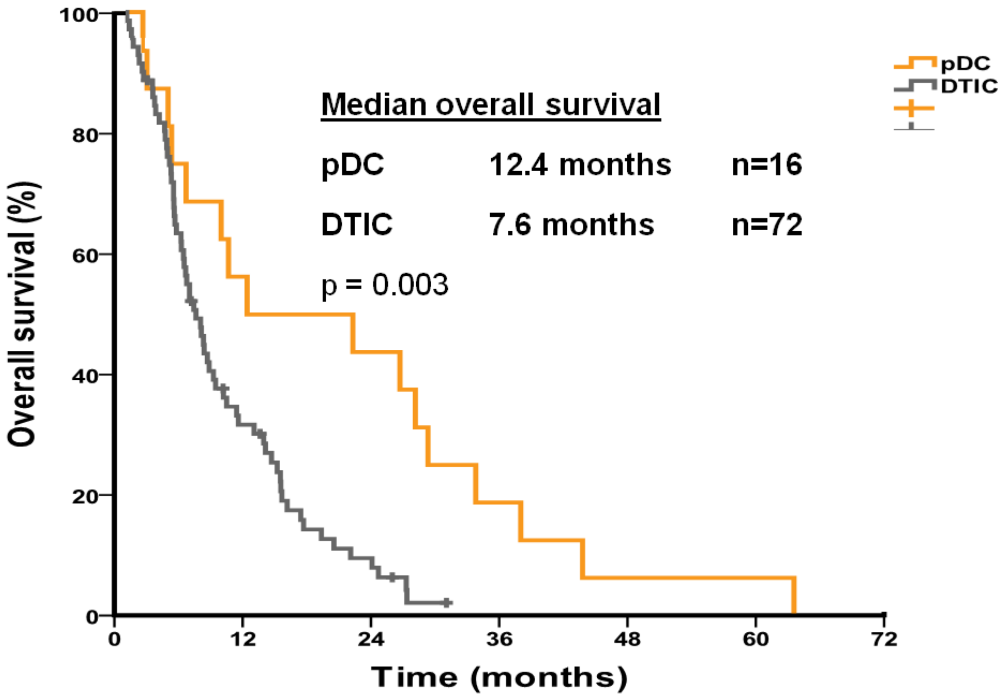

Figure 3. pDC vaccination improves overall survival in melanoma patients. Clinical outcome to pDC vaccination was compared to a group of matched historical control patients who received dacarbazine as first line treatment. Median overall survival for pDC vaccination was 12.4 months versus 7.6 months in the matched controls (p=<0.01). The median progression free survival did not show a significant difference (4.0 months versus 2.1 months).

Additionally, we performed a clinical trial in stage IV melanoma patients with myDC (Schreibelt et al, accepted CCR). Again, promising tumor-specific T cell responses and clinical responses were observed (Figure 4 and 5). The presence of T cell responses coincided with long-term progression-free survival (Figure 6). In our ongoing studies in melanoma patients (NL49528.000.14) and prostate cancer patients (NL491.43.000.14) vaccinated with myDC and pDC simultaneously, we found myDC-induced KLH-specific responses (Figure 7A) and pDC-induced IFN responses (Figure 7B).

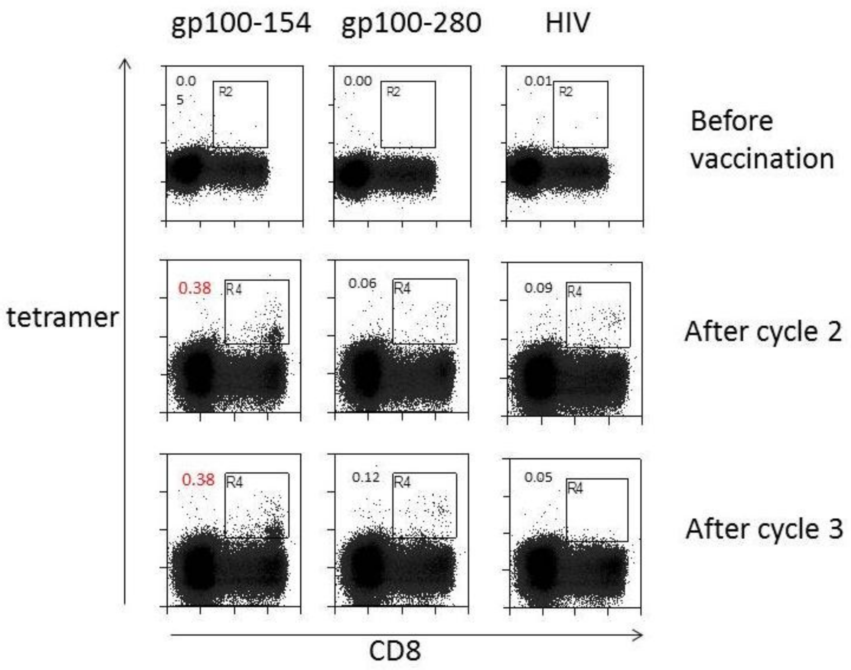

Figure 4. myDC vaccination induces tumor-specific immune responses in melanoma patients. Tetramer analysis by flow cytometry of peripheral blood mononuclear cells (PBMC) of a melanoma patient before vaccination and after two and three cycles of myDC vaccination demonstrates induction of gp100-specific CD8^{+} T cells by myDC vaccination.

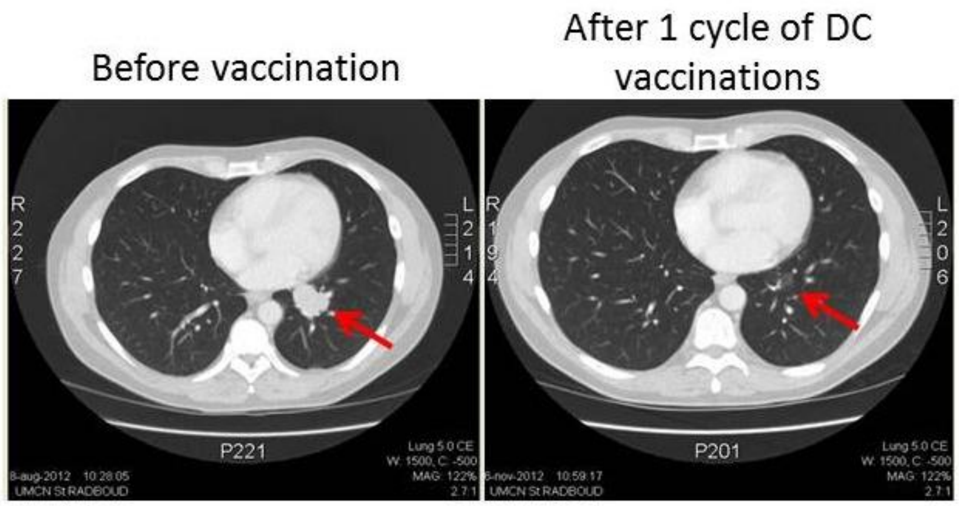

Figure 5. Reduction of tumor mass after DC vaccination. Computed tomography (CT) scan of the thorax of a melanoma patient before (left) and after (right) one cycle of three vaccinations with peptide-loaded myDC, clearly showing a strong reduction of a pulmonary metastasis.

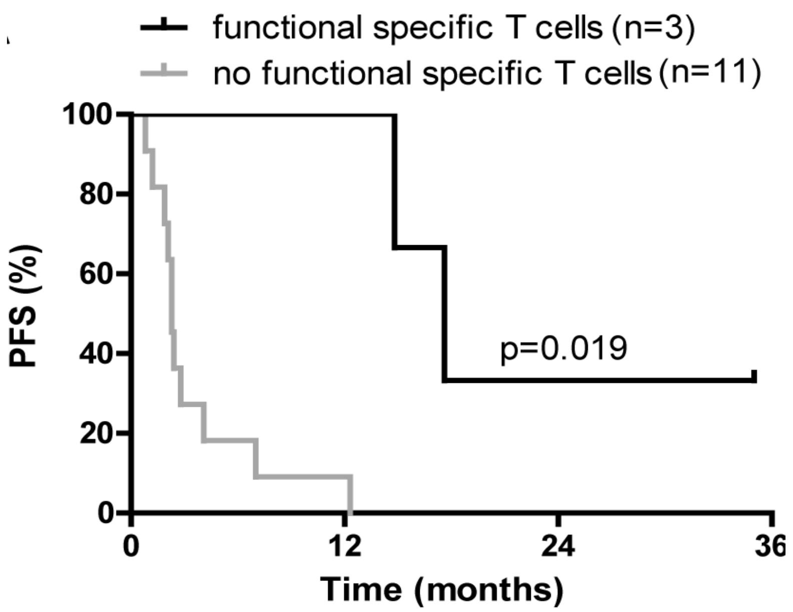

Figure 6. Correlation between presence of functional T cells and clinical outcome. Kaplan-Meier analysis of progression free survival according to the presence of functional tumor antigen-specific T cells. Median progression-free survival of patients with functional specific T cells was 17.6 months versus 2.3. For patients without tumor-specific T cells ($p<0.05$). The median overall survival did not show a significant difference (29.0 months versus 10.9 months).

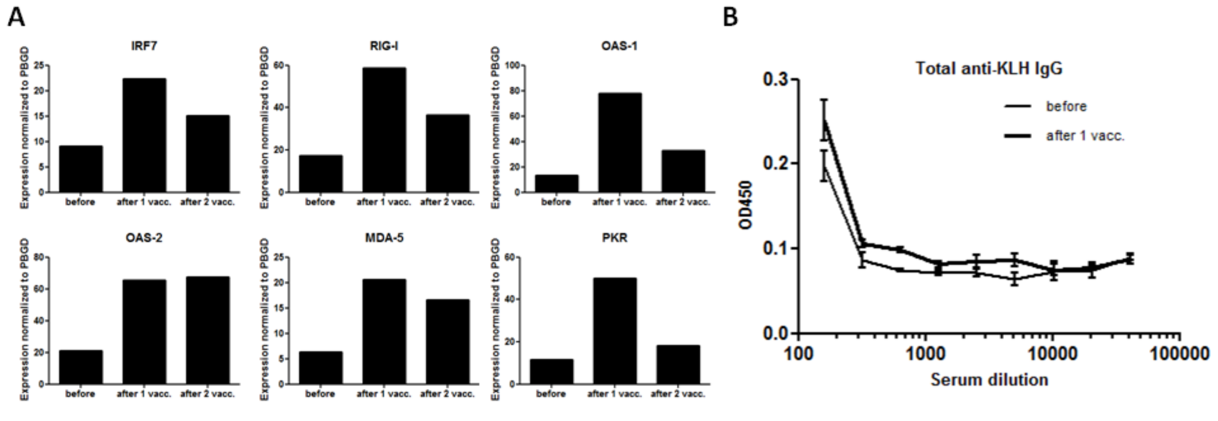

Figure 7. Simultaneous injection of myDC and pDC induces responses in melanoma patients. A. pDC-induced IFN-signature after vaccination with pDC and myDC. The graphs show gene expression levels of IFN-stimulated genes in the blood relative to PBGD expression before and after the first and second vaccination. B. Elevated levels of total IgG anti-KLH antibody titers after the first vaccination compared to before vaccination. It is anticipated that anti-KLH antibodies will further increases after subsequent vaccinations.

From our previous and ongoing studies with nDC we can conclude that was safe, with little treatment-related side effects. The most common side effects of DC vaccination are depicted in the table below.

Toxicity	pDC (n=15)	myDC (n=14)	pDC+myDC (n=2*)
Flu-like symptoms grade 1	13% (2)	29% (4)	0% (0)
Flu-like symptoms grade 2	0% (0)	7% (1)	0% (0)
Injection site reaction grade 1	13% (2)	0% (0)	0% (0)
Injection site reaction grade 2	0% (0)	0% (0)	0% (0)

* ongoing

Taken together, we demonstrated that it is feasible to use pDC and myDC and that these cells can be activated and loaded with peptides before injection into melanoma patients. Based on in vitro data and clinical studies, we hypothesize that the combination of myDC and pDC may induce stronger anti-tumor immune responses as compared to pDC or myDC alone, or moDC. Based on our experience, we believe that it is feasible to use pDC and myDC and that these cells can be activated and loaded with peptides before injection into melanoma patients.

Of all cancer types, melanoma is by far the most studied cancer type in DC immunotherapy. Schadendorf et al conducted the first, and thus far only, randomized DC vaccination trial in 2000 which did not show survival benefit compared to dacarbazine.[51] In the previous two decades numerous small, non-randomized, monocentrical trials with DC vaccination have been performed to optimize the DC vaccine and the first DC-like vaccine (sipuleucel-T in men with metastatic castration-resistant prostate cancer) has shown benefit on OS and is approved as standard treatment. After the decades of optimization of DC vaccines it is of importance to re-explore DC vaccination in a randomized fashion in melanoma.

2. Objectives of the study

2.1 Primary objective

To determine whether adjuvant treatment with nDC vaccination, after standard treatment in stage IIIB and IIIC melanoma patients, improves RFS as compared to treatment with matching placebo.

2.2 Secondary objectives

- To determine whether post-operative adjuvant nDC vaccination improves 2-year and median OS as compared to placebo.
- To compare adverse event (AE) profiles between patients receiving nDC vaccination versus patients receiving treatment with matching placebo.
- To determine immunological responses in patients receiving nDC vaccination (arm A) and placebo (arm B).
- To determine factors, genetic and microbiome, responsible for the variability of immune responses in patients receiving nDC vaccination (arm A) and placebo (arm B).
- To compare the Quality of Life (QoL), Quality Adjusted Life Years (QALY) and health economic aspects between nDC vaccination and placebo.

2.3 End-points

Definitions of endpoints can be found in chapter 7.

2.3.1 Primary endpoint

- 2-year RFS rate

2.3.2 Secondary endpoints

- 2-year OS rate, OS and RFS duration
- AE profiles (see chapter 7)
- Immunological responses (see chapter 7)
- QoL/QALY (see chapter 9)
- Costs (see chapter 10)

3. Study population

3.1 Number of subjects and patient recruitment

Two hundred ten patients will be randomized 2:1 to nDC vaccination (n=140) versus placebo (n=70) using a minimization procedure accounting for the following stratification factors:

- Stage of the disease (stage IIIB versus stage IIIC)
- Adjuvant radiotherapy (planned/received versus not planned/not received)
- BRAF mutation status (BRAF wild type versus BRAFV600 mutation (versus unknown))
- HLA-type (HLA-A2 negative versus HLA-A2 positive)
- nDC production centre (Nijmegen versus Amsterdam)

We estimate that 300 patients per year are newly diagnosed with stage IIIB or IIIC melanoma in the Netherlands (6% of total new melanoma patients in the Netherlands (n=4650 a year) are stage IIIB or stage IIIC).[52] For follow-up after surgery, this group of patients often remains in peripheral medical centers due to the lack of a generally accepted adjuvant treatment. The physicians at the participating hospitals (Free university medical centre Amsterdam (VUmc), Netherlands Cancer Institute (NKI) Erasmus Medical Centre Rotterdam (Erasmus MC) and Isala clinics Zwolle) are in close contact with these centers (consultancy function and regional reference centers for metastatic melanoma) and thereby will actively recruit patients to their centers. Partner hospitals from the Radboud university medical centre Nijmegen (Radboudumc) already closely collaborate by weekly video consultation. The participating centers are designated melanoma centers. It is expected that a small number of eligible patients will refuse to participate in this study.

Our institution, Radboudumc, is a regional reference centre for melanoma patients with approximately 35 stage IIIB and IIIC melanoma patients each year. Radboudumc expects to recruit 25 patients per year during the accrual period (36 months). The number of stage IIIB and IIIC patients seen in the other 3 participating centers are larger or comparable to Radboudumc. Due to their lack of experience with the nDC vaccinations (logistical complexity) their estimated number of patients to be included in this study is rather conservative, 15 patients per centre per year. Six months after start of study, we will consult all centers whether the expected numbers will be reached. We have already actively

recruited referral centers, predominantly the partner hospitals of Radboudumc. If patient inclusion is still less than required for successful completion of the study we will actively promote the study in referral hospitals in other parts of the Netherlands.

Justification of placebo

In stage III melanoma there is no standard adjuvant treatment after surgery, the current standard of care consists of follow-up only. To prevent a beneficial effect in a patient due to the patient's expectations concerning the DC administration rather than from the nDC itself, patients receiving placebo will undergo the same treatment-related proceedings as patients in the treatment arm. In this study the sham treatment consists of one leukapheresis, intranodal injections, intradermal injections and skin biopsies. Although rather invasive for placebo patients, we believe this is warranted because patients and treating physicians will not be able to distinguish between the two treatment arms based on side effects. It is anticipated that nDC have very mild side effects, flu-like symptoms grade 1 in 30% of the patients and in 15% injections side reactions.
In this study the placebo will consist of NaCl supplemented with Albuman. Alternative options, like immature DC or peptides only are not considered inactive substances. Both immature DC and peptides might have even have a negatively influence on the disease since they are able to induce immune tolerance. Injection of mature pDC or myDC without peptides might result in a positive effect due to the production of soluble factors like cytokines by the two cell types. pDC produce high amounts of type I IFN which are highly effective against virus infections and possibly also against tumors since IFN-treatment is approved by the FDA for treatment of stage III melanoma patients due to its positive effect on the disease. myDC produce high amounts of IL-12, a cytokine known to have a beneficial effect on the skewing of T helper responses towards anti-tumor help, and therefore injection of these cells is also not considered inactive.

3.2 Patient eligibility criteria

In order to be eligible to participate in this study, a subject must meet all of the following criteria:
- At least 18 years of age.

- Histologically confirmed stage III cutaneous melanoma, classified as stage IIIB or IIIC disease (AJCC 2009). Patients with completely resected in-transit and/or satellite metastases and patients with unknown primary melanoma are allowed in this trial.

For the purpose of staging for eligibility, macrometastases are defined as:

i. A palpable node (confirmed as malignant by pathology)
ii. A non-palpable but enlarged lymph node by CT (at least 15mm in short axis) and confirmed as malignant by pathology
iii. A PET positive lymph node of any size confirmed by pathology
iv. Evidence of pathologically macrometastatic disease in one or more lymph nodes defined by one or more foci of melanoma at least 1cm in diameter

- RLND of involved site with complete resection of melanoma as documented on the operating report and pathology report with at least the minimal levels excised as stated in national guidelines and at least 15 excised lymph node in case of RLND of the neck; at least 10 excised lymph nodes in case of RLND of the axilla, or at least 5 excised lymph nodes in case of inguinal RLND; or sentinel node procedure (SNP) showing limited sentinel-node positive disease without an indication for RLND.
- RLND or SNP must be performed within 12 weeks prior to start of study (leukapheresis).
- Recovered from definitive surgery (e.g. no uncontrolled wound infections or indwelling drains).
- Absence of distant metastases must be documented by a CT scan of the chest and abdomen (including the pelvis) or a Positron Emission Tomography (PET) scan, the scan should have been performed within 6 weeks prior to inclusion, before or after surgery. In addition, a physical exam after surgery must be performed also excluding distant metastases.
- No clinical evidence for brain metastases. If brain metastases are clinically suspected, a CT or Magnetic Resonance Imaging (MRI) scan of the brain must exclude brain metastases.
- World Health Organization (WHO) performance status of 0 or 1 at time of randomization (see appendix 1).
- Adequate hematologic, renal and liver function as defined by laboratory values performed within 4 weeks prior to randomization:
    - White Blood Cell count (WBC) > 3.0 x 10^9/L
    - Absolute Lymphocyte Count (ALC) > 0.8 x 10^9/L

- platelet count > 100 x 10^9/L
- serum creatinine (creat) < 150 µmol/L
- serum bilirubin < 25 µmol/L
- serum LDH ≤ Upper limit of laboratory normal range (ULN)

- No second malignancy in the previous 5 years, with the exception of adequately treated carcinoma in-situ and basal or squamous cell carcinoma of the skin.
- No concomitant use of immunosuppressive drugs orally or intravenously. Topical and intranasal steroids are permitted.
- No uncontrolled infectious disease, i.e. negative testing for HIV, Hepatitis B Virus (HBV), Hepatitis C Virus (HCV) and syphilis (Treponema Pallidum Hemagglutination Assay (TPHA)).
- No autoimmune disease such as, but not limited to, inflammatory bowel disease, multiple sclerosis and lupus. Patients with type 1 diabetes mellitus, hypothyroidism after autoimmune thyroiditis and skin disorders are not excluded.
- No serious (bleeding and clotting) condition that may interfere with safe leukapheresis.
- No pregnant or lactating women.
- No Women Of Child-Bearing Potential (WOCBP) and male participants with a female partner of child-bearing potential who are unwilling or unable to use an acceptable method to avoid pregnancy for up to 8 weeks after the last administration of the treatment. WOCBP include any female who has experienced menarche and who has not undergone successful surgical sterilization (hysterectomy, bilateral tubal ligation or bilateral oophorectomy) or is not postmenopausal [defined as amenorrhea > 12 consecutive months].
- Patients must have absence of any psychological, familial, sociological or geographical condition potentially hampering compliance with the study protocol and follow-up schedule; those conditions must be discussed with the patient before registration in the trial.
- Expected adequacy of follow-up.
- Written informed consent.

4. Study design

This phase 3 study will be a double-blind, multicentre, randomized, placebo-controlled trial designed to assess the efficacy of nDC vaccination as adjuvant therapy for patients with stage IIIB or stage IIIC cutaneous melanoma after surgical resection. A total of 210 evaluable patients will be randomized between nDC vaccination (arm A) and placebo (arm B) in the ratio 2:1. The 2:1 ratio was chosen to grant the wish of the patients and patient organization to have a higher change of getting study treatment compared to placebo. Patients will be randomized and leukapheresis must start within 1 week of randomization and within 12 weeks after RLND or SNP. Randomization will be performed centrally using a minimization procedure accounting for the following stratification factors:

- Stage of the disease (stage IIIB versus stage IIIC)
- Adjuvant radiotherapy (planned/received versus not planned/not received)
- BRAF mutation status (BRAF wild type versus BRAF V600 mutation (versus unknown))
- HLA-type (HLA-A2 negative versus HLA-A2 positive)
- nDC production centre (Nijmegen versus Amsterdam)

The treatment will be continued for a maximum of 1.5 years (9 study drug administrations) or until recurrence of disease, unacceptable toxicity or withdrawal from the study (due to patient refusal or patient's best interest to stop according to treating physician). The disease will be assessed at baseline, then every 3 months for the first 1.5 years after of the study (treatment phase) and every 3-6 months in the following 3.5 years (follow-up phase). Toxicity will be assessed on every visit.

For specific details on statistical considerations see chapter 8.

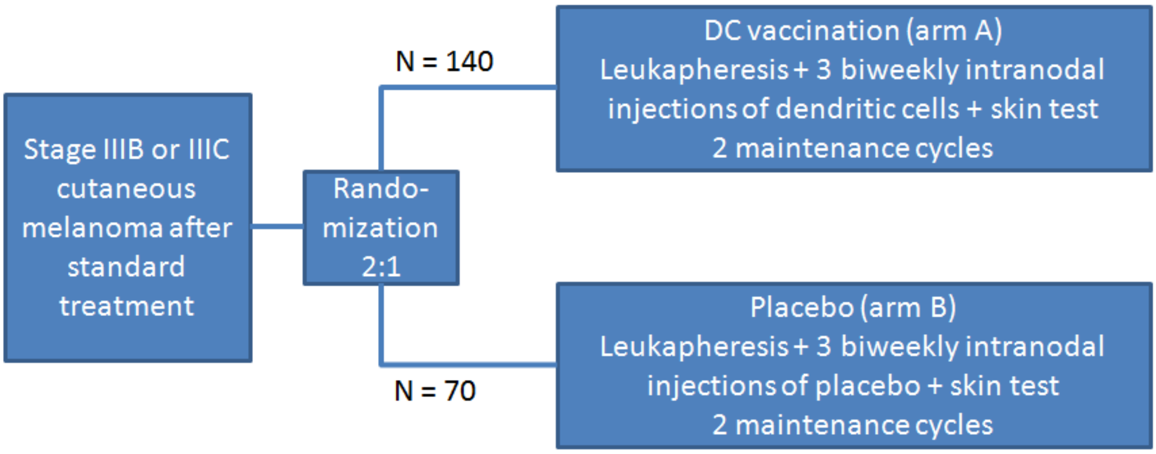
Stage IIIB or IIIC cutaneous melanoma after standard treatment
Rando-mization 2:1
N = 140
DC vaccination (arm A)
Leukapheresis + 3 biweekly intranodal injections of dendritic cells + skin test
2 maintenance cycles
N = 70
Placebo (arm B)
Leukapheresis + 3 biweekly intranodal injections of placebo + skin test
2 maintenance cycles

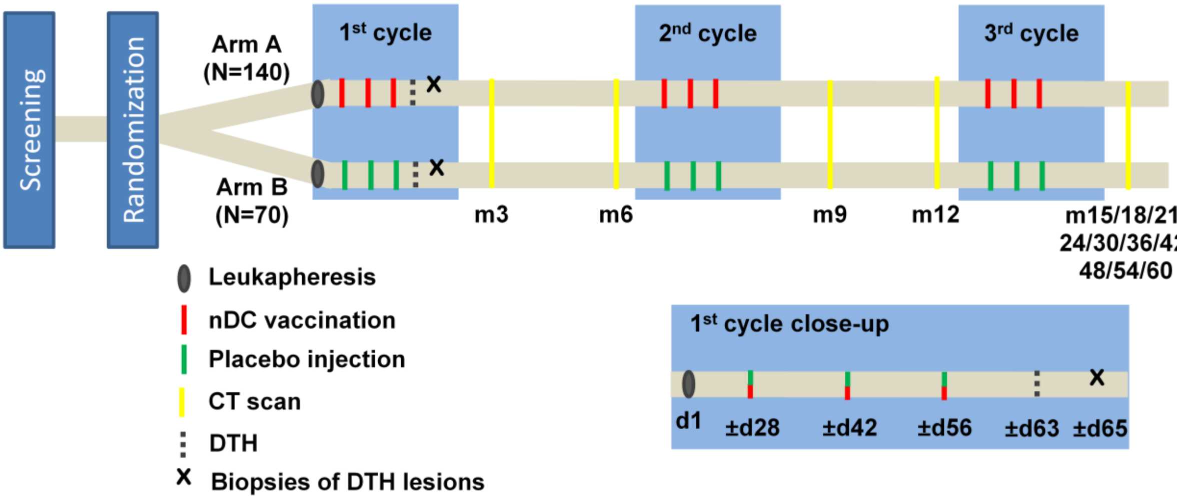
Screening
Randomization
Arm A (N=140)
Arm B (N=70)
1st cycle
2nd cycle
3rd cycle
m3
m6
m9
m12
m15/18/21/24/30/36/42/48/54/60
Leukapheresis
nDC vaccination
Placebo injection
CT scan
DTH
Biopsies of DTH lesions
1st cycle close-up
d1
±d28
±d42
±d56
±d63
±d65

5. Therapeutic regimens, expected toxicity, dose modifications

5.1 Investigational treatment

All eligible and consenting stage IIIB and IIIC melanoma patients will undergo a leukapheresis at the start of the first cycle of treatment. From this autologous blood product, nDC will be isolated for patients in arm A or the blood product will be stored/used for melanoma/immunotherapy research (consented patients in arm B). Vaccinations are done with pDC and myDC loaded with tumor peptides, prepared according to the IMPD "Natural dendritic cell product". The quality control standards and requirements for this product are described in this dossier, and the required approvals have been obtained.

Delayed-type hypersensitivity (DTH) skin tests and biopsies from DTH sites will be performed 1-2 weeks after the third study drug administration in cycle 1. Induration will be measured after 48 hours and punch biopsies will be taken from each DTH site (6mm) and normal skin (3mm). Patients in the nDC vaccination arm (arm A) will receive 3 nDC injections intranodally ($8x10^6$ nDC) and 4 DTH challenges with nDC ($0.5x10^6$ nDC) intradermally injected at the back of the patient. Patients in the placebo arm (arm B) will receive 3 placebo injections intranodally and 4 DTH challenges with placebo intradermally injected at the back of the patient. If patients remain clinically free of melanoma, this cycle will be repeated twice with 6 month intervals, consisting of nDC injections only. For details about analyses of obtained materials, including skin biopsies, see appendix 2.

Immune-related AE may be seen in nDC vaccinated patients, mainly consisting of flu-like symptoms and injection-site reactions. The absence of immune-related AE does not exclude patients being treated with nDC vaccination as it is expected that approximately only 30% of patients who receive nDC vaccination will develop mild immune-related symptoms. **Patients are not to be taken off protocol due to the incorrect assumption that the absence of immune-related AE implies that the patient is receiving placebo.**

5.2 Dose and drug administration

Patients in arm A will receive 8 million nDC reconstituted in maximal 0.2 ml injection liquid whereas patients in arm B will receive maximal 0.2 ml injection liquid only (matching placebo), which consists of NaCl 0.9% supplemented with 25% Albuman (final concentration). If not enough nDC are obtained from leukapheresis (for patients in arm A) for the vaccination of 8 million nDC per vaccination, a minimum of 3 million nDC will be given per vaccination, with subsequent placebo injections where necessary.
Based on our experience, we are able to make a nDC vaccine meeting the release criteria (see IMPD) in over 95% of patients who have had leukapheresis. If in a patient we are not able to make a nDC vaccine meeting the release criteria, patient will be given placebo but remains in the study treatment arm (arm A).
Liquids, with (arm A) or without (arm B) nDC will be injected intranodally under ultrasound guidance biweekly. Intranodal injection of nDC will be given into an intact, clinically tumor-free lymph node based on ultrasound aspects in either site, due to practical reasons, preferable the groin. The site of RLND and SNP is excluded, e.g. patients who had a RLND of the left groin can be vaccinated in the right groin (preferable) or either axillae. The nDC vaccine or placebo may, but does not need to be, injected in the same node upon consecutive study drug administrations.

5.3 Patient monitoring

The patients will be observed for 30 minutes after intranodal administration of nDC or placebo, based on previous experience (see below).

5.4 Dose adjustments

Based on the experience with our intranodally injected nDC in metastatic melanoma patients, we expect that the intranodally injected nDC will be well tolerated. Expected AE are mild flu-like symptoms and injection-site reaction, grade 1 or incidentally grade 2 as described by the Common Terminology Criteria for Adverse Events (CTCAE).
Treatment should be withheld for any grade 3 or 4 (CTCAE criteria) or otherwise intolerable toxicity that cannot be adequately managed with optimal supportive care, if considered

related to the study drug. Treatment may be resumed once the toxicity improves to CTCAE grade 1 or baseline, or in the case of a skin rash or skin hypopigmentation (vitiligo) to CTCAE grade 2. Treatment can resume at the same dose, especially in case of brief grade 3 or 4 fever or flu-like symptoms. However, if this is not likely to be tolerated at the discretion of the Investigator then the dose should be reduced to 50%. If the drug is not tolerated at this level then the drug should be discontinued. N.B. once a dose reduction has occurred then the patient must remain at that dose and not be increased back to a previous dose level. Hypersensitivity to nDC vaccination due to Keyhole limpet hemocyanin (KLH) is possible but extremely rare, and may present with hypotension, facial flushing, back pain, dyspnoea, laryngeal and facial oedema and urticaria. Emergency management should be in line with local policy. If a severe hypersensitivity reaction against the nDC vaccination occurs, the drug will be discontinued.

5.5 Duration of therapy

Patients will receive 3 biweekly nDC vaccinations or placebo per cycle. If patients remain clinically free of melanoma, this cycle will be repeated twice with 6 month intervals. Patients may discontinue protocol treatment when unacceptable toxicity is encountered, if patients develop an intercurrent illness which would in the judgment of the Investigator affect patient safety, the ability to deliver treatment or the primary study endpoints, or at patient's request.

5.6 Concomitant therapy

During treatment in this study, the use of systemic corticosteroids and non-steroidal anti-inflammatory drugs are strongly discouraged because of their immunosuppressive effect, except in case of life-threatening toxicity. Any other investigational agents, as well as previous immune therapy, are also prohibited during study duration. Other drugs, including anti-histaminica for allergic reactions and paracetamol for headache, muscle-ache or flu-like symptoms are allowed. Adjuvant post-operative radiotherapy following national guidelines is allowed.

6. Clinical evaluation, laboratory tests, follow-up

6.1 Study procedures before randomization (screening)

The patient's written informed consent for clinical trial participation must be given prior to the performance of any screening/protocol procedures that are not part of national routine guidelines.

The following procedures will be performed during the screening phase and results should be known before randomization:

- Eligibility criteria (see chapter 3)
- History and physical examination
- HLA typing
- Viral testing including HIV, HBV, HCV, EBV, CMV, HTLV1/2 serology and TPHA (within 28 days of leukapheresis)
- WHO performance status
- Weight, length and vital signs
- Laboratory tests: hemoglobin (Hb), hematocrit (Ht), WBC, ALC, platelets, creat, urea, bilirubin, aspartate-aminotransferase (AST), alanine-aminotransferase (ALT), alkaline phosphatase (AP), LDH, albumin, C-reactive protein (CRP), sodium (Na), potassium (K), calcium (Ca), phosphatase (P) and y-glutamyl transferase (GGT)
- BRAF V600 mutation analyses
- Concomitant medications assessment
- CT of the chest and abdomen including pelvis (and a CT of the neck for head and neck primaries) or PET scan are mandatory for the exclusion of distant metastases and must be performed within 6 weeks before inclusion, before or after surgery.
- Upon clinical suspicion of brain metastases, a CT or MRI of the brain is mandatory and must not show any signs of metastases.
- QoL questionnaires, within 2 weeks of randomization (see chapter 9)
- The agreement on the use of an adequate birth control method by patients of childbearing/ reproductive potential until 8 weeks after the last administration of the study drug should be documented in the patient record.

On exception, screening procedures, including viral testing, laboratory tests, HLA-typing and CT scan, can be performed elsewhere.

6.2 Study procedures during treatment phase (first 1.5 years)

The following procedures will be performed during the treatment phase (for days see summary table in section 6.4):

- History and physical examination
- WHO performance status
- Toxicity assessment
- Weight and vital signs
- Laboratory tests: Hb, Ht, WBC, ALC, platelets, creat, urea, bilirubin, AST, ALT, AP, LDH, albumin, CRP, Na, K, Ca, P and GGT. On days with administration of the study drug, the blood has to be drawn before vaccination, but the results may be awaiting when the study drug is injected, provided there is no reason for which the results have to be awaited at the discretion of the investigator.
- Concomitant medications assessment
- QoL questionnaires (see chapter 9)
- Blood in heparin tubes(for immunomonitoring; see appendix 2). On days with administration of the study drug, the blood has to be drawn before vaccination.
- Serum (for immunomonitoring; see appendix 2) . On days with administration of the study drug, the blood has to be drawn before vaccination.
- Stool samples, the day before or on the day of administration of the study drug for microbiome analyses
- Swab samples of skin and mouth for microbiome analyses
- Blood in PAXgene tubes, one before and one 2-4 hours after study drug administration 3 (Week 9; for immunomonitoring; see appendix 2)
- DTH skin test and biopsies (for immunomonitoring; see appendix 2)
- Request archival tumor tissue for biomarker analyses
- CT of the chest and the abdomen including pelvis (and a CT of the neck for head and neck primaries) every 3 months

- Data collection for cost analyses

6.3 Study procedures during the follow-up phase (last 3.5 years)

The following procedures will be performed during the follow-up phase (for days see summary table in section 6.4:

- History and physical examination
- WHO performance status
- Toxicity assessment
- Weight and vital signs
- Laboratory tests: Hb, Ht, WBC, ALC, platelets, creat, urea, GGT, bilirubin, AST, ALT, AP, LDH, albumin, CRP, Na, K, Ca and P.
- QoL questionnaires
- Blood in heparin tubes
- Serum
- CT of the chest and the abdomen including pelvis (and a CT of the neck for head/neck primaries) every 3-6 months
- Request archival tumor tissue for biomarker analyses
- Data collection for (cost) analyses, including data collection on subsequent treatments after recurrence of disease

6.4 Summary tables

Screening period

Procedure	Screening visit Day -28 till Day 0	Notes
**Eligilibility assessments**		
Informed Consent	X	
Eligibility criteria	X	
HLA-subtyping	X	
History	X	
**Safety assessments**		
Physical exam	X	
Vital signs, weight, height	X	Vital signs: blood pressure, heartrate
WHO performance score	X	
Laboratory tests	X	Hb, Ht, WBC, ALC, platelets, Creat, urea, GGT, bilirubin, AST, ALT, AP, CRP, albumin, LDH, Na, K, Ca, P
BRAF mutation analyses	X	
Medication assessment	X	
Viral testing + TPHA	X	Including HIV, HBV, HCV, CMV, EBV (HTLV-1 and 2) serology
**Efficacy assessments**		
QoL questionnaires	X*	Including EQ-5D, EQ- 5D VAS, QLQ-C30, FACT-M, QoL MIND-DC, Short Health and labour
CT chest/abdomen/pelvis	X	Allowed pre-surgery as routine guidelines; PET scan is allowed instead of CT.<br>CT is including neck for head and neck primaries

* within 2 weeks of randomization

Treatment phase – Cycle 1

Procedure	Day 1 Week 1	Day 1 Week 5	Day 1 Week 7	Day 1 Week 9	Day 1 Week 10	Day 3 Week 10	Day 1 Week 14	Day 1 Week 26
Windows[a]		±7dy	±3dy	±3dy	-2/+7dy	+1dy	±7dy	±7dy
Leukapheresis	x							
Study drug		1	2	3				
CT scan[b]							x	x
Physical exam		x	x[c]	x[c]	x		x	x
Vital signs, weight		x			x		x	x
WHO score		x			x		x	x
Toxicity assessment		x	x	x	x		x	x
Laboratory tests		x	x	x	x		x	x
Medication assessment		x	x	x	x		x	x
Heparin blood	80 ml[d]			80 ml[d]	80ml[d]			
Viral testing	x[e]							
Serum	5 ml[d]			5 ml[d]	5 ml[d]			
PAXgene blood				2x 2,5 ml[f]				
Stool		x		x				
Skin swab		x		x				
Mouth swab		x		x				
DTH skin test					x			
Skin biopsies						x		
QoL questionnaires[g]							x	x
Cost analyses							x	x

[a] windows of subsequent vaccinations or DTH skin test are calculated from the previous vaccination of the cycle (e.g. if vaccination 1 ig given om day 1 week 6, vaccination 2 should be scheduled on day 1 week 8 ±3 days) , the window of the skin biopsies is calculated from

the DTH skin test (biopsies are taken 2 or 3 days after the DTH skin test); windows of FU days, e.g. Day 1 week 14, are not dependent on previous visits.

[b] CT chest/abdomen/pelvis, including neck for head and neck primaries

[c] if clinically indicated

[d] drawn before administration of the study drug

[e] Viral testing includes HIV, HBV, HCV and TPHA

[f] one tube drawn before administration of the study drug and one tube 2-4h afterwards

[g] QoL questionnaires must be filled out within a week before, or at the hospital, when the patient comes for a scheduled visit. If the patient did not fill the questionnaires out before the visit, they will be asked to fill it out at the hospital or, if not feasible, at home as soon as possible, but no later than 5 days after the visit.

[h] Only SAE that are believed to be related to prior treatment with the study drug.

Treatment phase – Cycle 2

**Procedure**	**Day 1 Week 31**	**Day 1 Week 33**	**Day 1 Week 35**	**Day 1 Week 39**	**Day 1 Week 52**
Windows[a]	±2wk	±3dy	±3dy	±7dy	±7dy
Study drug	4	5	6		
CT scan[b]				x	x
Physical exam	x	x[c]	x[c]	x	x
Vital signs, weight	x			x	x
WHO score	x			x	x
Toxicity assessment	x	x	x	x	x
Laboratory tests	x	x	x	x	x
Medication assessment	x	x	x	x	x
Heparin blood	80 ml[d]			80 ml[d]	
Serum	5 ml[d]			5 ml[d]	
Stool	x				
Skin swab	x				
Mouth swab	x				
QoL questionnaires[g]				x	x
Cost analyses				x	x

Treatment phase – Cycle 3

Procedure	Day 1 Week 57	Day 1 Week 59	Day 1 Week 61	Day 1 Week 65	Day 1 Week 78	Day 1 Week 91
Windows[a]	±2wk	±3dy	±3dy	±7dy	±7dy	±7dy
Study drug	7	8	9			
CT scan [b]				x	x	x
Physical exam	x	x[c]	x[c]	x	x	x
Vital signs, weight	x			x	x	x
WHO score	x			x	x	x
Toxicity assessment	x	x	x	x	x[h]	x[h]
Laboratory tests	x	x	x	x	x	x
Medication assessment	x	x	x	x	x	x
Heparin blood	80 ml[d]			80 ml[d]	80 ml[d]	
Serum	5 ml[d]			5 ml[d]	5 ml[d]	
Stool				x		
Skin swab				x		
Mouth swab				x		
QoL questionnaires[g]				x	x	
Cost analyses				x	x	

Follow-up phase

	Month 24	Month 30	Month 36	Month 42	Month 48	Month 54	Month 60
Windows	±2wk	±2wk	±2wk	±2wk	±2wk	±2wk	±2wk
CT scan[b]	x	x	x	x	X	x	x
Physical exam	x	x	x	x	X	x	x
WHO score	x	x	x	x	X	x	x
Toxicity assessment	x[h]	x[h]	x[h]	x[h]	x[h]	x[h]	x[h]
Laboratory tests	x	x	x	x	X	x	x
Heparin blood	80 ml						80 ml
Serum	5 ml						5 ml
QoL questionnaires[g]	x		x				x
Cost analyses	x		x				x

7. Criteria of evaluation

7.1 Primary endpoint

The primary endpoint is 2-year RFS rate, defined as the percentage of patients who are alive and without recurrence of melanoma 2 years after randomization.

7.2 Secondary endpoints

7.2.1 Secondary efficacy endpoints

OS is defined as the interval from randomization to the date of death, irrespective of the cause of death; patients still alive will be censored at the date of the last assessment. Both 2-year OS rate and the median OS duration will be assessed. Five year after inclusion of all patients, a long term follow-up will be conducted for OS. Data on OS and subsequent treatments for recurrence of disease will be collected during study follow-up and thereafter. RFS is defined as the time from randomization to either the date of first recurrence of melanoma or the date of death, whichever occurs first. RFS will be assessed by physical examination and CT of the chest and abdomen every 3-6 months, or on clinical indication. Both the presence of loco-regional and distant metastases are considered as recurrence. The first date when recurrence was observed/confirmed, either by imaging or by pathology, is taken into account. If recurrence is only observed by imaging, and not confirmed by pathology, an radiologist should confirm the presence of recurrent disease as soon as possible. Any death occurring without prior documentation of tumor recurrence will be considered as an event (and will not be censored in the statistical analysis) as this approach is less prone to introduce bias. If no event (any recurrence or death) has occurred by the time of the analysis, then the time to event will be censored at the date of the last assessment of the patient in question. Any new primary cancer at another site, including second primary melanoma, will not be considered as an event for RFS analysis. Median RFS duration will be assessed.

7.2.2 Secondary safety and tolerability endpoints

Toxicity will be assessed using the Common Toxicity Criteria for Adverse Events version 4.03 (a link to the CTCAE can be accessed from the European Organization for Research and Treatment of Cancer (EORTC) web site http://www.eortc.be/). All AE should be collected from signing informed consent up to 30 days after the last administration of study treatment or start of another cancer therapy, whichever occurs first.

All AE will be recorded on the case report forms; the Investigator will decide if those events are drug related (not related, not likely, possibly, probably, certainly) and this decision will be recorded on the forms for all AE. Serious AE (SAE) are defined by the Good Clinical Practice Guideline. SAE must be immediately reported according to the procedure detailed in the protocol (see chapter 13).

After the period of 30 days after the last administration of study treatment or after start of another cancer therapy, Investigators should report any deaths or SAE that are believed to be related to prior treatment with the study drug.

Immune-related AE may be seen in nDC vaccinated patients, mainly consisting of flu-like symptoms and injection-site reactions. The absence of immune-related AE does not exclude patients being treated with nDC vaccination. **Patients are not to be taken off protocol due to the incorrect assumption that the absence of immune-related AE implies that the patient is receiving placebo.**

7.2.3 Secondary quality of life and cost effectiveness endpoints

To measure the quality of health status of the patients a validated Health-Related Quality of Life (HRQoL) instrument will be used, the EuroQol-5D (EQ-5D). This HRQoL instrument will be completed by the patients and is available in a validated Dutch translation (see appendix 3). The EQ-5D is a generic HRQoL instrument comprising five domains: mobility, self-care, usual activities, pain/discomfort and anxiety/depression. The EQ-5D index is obtained by applying predetermined weights to the five domains. This index gives a societal-based global quantification of the patient's health status on a scale ranging from 0 (death) to 1 (perfect health). Patients will also be asked to rate their overall HRQoL on a visual analogue scale (EQ- 5D VAS) consisting of a vertical line ranging from 0 (worst imaginable health status) to 100 (best imaginable). EQ-5D utilities will be combined with survival data using the

trapezium rule resulting in QALY. Furthermore the EORTC Quality of Life Questionnaire (QLQ-C30), which is a questionnaire developed to assess the quality of life of cancer patients, (see appendix 4), a melanoma-specific questionnaire (FACT-M; see appendix 5), and a study-specific questionnaire (QoL-MIND-DC; see appendix 6) will be assessed as well. Timing according to the summary table (6.4). Results from the EORTC QLQ-C30 will be reported, besides the Incremental Cost-Effectiveness Ratios (ICER).

Primary outcome measures for the economic evaluation, considering both the trial follow-up period and modeling time frame, are costs (direct and indirect) and QALY/quality adjusted survival. Input for the modeling part stems largely from the trial completed with information from earlier studies and secondary data. The ICER "cost per QALY gained" based on EQ-5D utilities will be computed and uncertainty will be determined using an appropriate method such as: the bootstrap method (empirical part) and 1st and 2nd order Monte Carlo simulation (modeling part). A cost-effectiveness acceptability curve will be derived that is able to evaluate efficiency by using different thresholds (willingness to pay) for a QALY. The impact of uncertainty surrounding deterministic parameters (for example prices) on the ICER will be explored using one-way sensitivity analyses on the range of extremes. Secondary ICER as 'cost per recurrence prevented' and 'cost per life year gained' will also be reported, in the same fashion as described above.

7.2.4 Immunological endpoints, biomarkers analyses and determination of factors responsible for the variability of immune responses

Immunological endpoints and biomarkers will be analyzed in both arms of the trial.

The immunological endpoints are:

- The occurrence and magnitude of functional responses and tetramer analysis of skin test-infiltrating T cells against tumor peptides.
- The occurrence and magnitude of functional responses and tetramer analysis of T cells in peripheral blood against tumor peptides.
- The occurrence and magnitude of type I IFN gene expression in PBMC shortly after study drug administration.

- The occurrence and magnitude of proliferative, effector cytokine- and humoral responses to KLH, an immunogenic providing T cell help.
- To assess the presence of a correlation between immunologically responding compared to immunologically non-responding nDC vaccinated patients (arm A) and the RFS and OS.

For details on immunomonitoring assays see appendix 2.

Biomarker specimens will be analyzed with the objective of identifying additional response prediction markers and biomarkers that explain mechanisms of immune resistance. For details on the collection of exploratory biomarker assessments in this study see appendix 2.

Genetic and microbiome factors will be determined with the objective to identify factors in melanoma patients responsible for the variability of immune responses upon nDC vaccination. These findings may have important consequences for immunotherapy. Inhibition of cytokines that are strongly influenced by the microbiome can potentially be modulated through diet, elimination of specific species, or fecal microbiota transplantation, host genomics-modulated cytokines may be more effectively targeted through inhibitory pharmacological approaches (e.g., neutralizing monoclonal antibodies). For details on the collection of exploratory genetic and microbiome factor assessments in this study see appendix 2.

8. Statistical considerations

8.1 Statistical design

8.1.1 Sample size

The primary objective of this study is to determine whether adjuvant nDC vaccination improves 2-year RFS rate as compared to placebo in patients with resected stage IIIB or IIIC melanoma. Based on previous results on adjuvant DC vaccination in stage IIIB and IIIC melanoma patients, we expect that 70% of the DC vaccinated patients will remain

recurrence free for 2 years (unpublished data). The anticipated 2-year RFS rate of the control arm is 50%.

For the comparison of RFS rate at 2 years between the two arms, with a 2-sided α=0.05 and power of 80%, a Cochran-Mantel-Haenszel test will be used.

According to calculations, a total of 210 patients must be included (140 patients in arm A and 70 patients in arm B) in this study, resulting in the following estimated timelines: Assuming that the entry rate will be, on average 70 patients per year (see section 3.1), the total accrual period will be 36 months. Five percent extra patients will be included to take into account a few patients who might be included accidentally based on ineligibility. Therefore a total of 221 patients will be randomized. If in a patient we are not able to perform a leukapheresis by a peripheral line, patient will be taken off study and will be replaced in the trial.

8.1.2 Randomization and stratification

Patients will be centrally randomized and stratified according to stage of the disease, adjuvant radiotherapy, BRAF V600 mutation status, HLA-type and nDC production centre. A minimization technique will be used for random treatment allocation stratifying by stage of the disease (IIIB versus IIIC), adjuvant radiotherapy (planned/received versus not planned/not received), BRAF mutation status (BRAF wild type versus BRAF V600 mutation (versus unknown)), HLA-type (HLA-A2 negative versus HLA-A2 positive) and nDC production centre (Nijmegen versus Amsterdam). This study will be double-blinded and placebo-controlled. Patients who are free from melanoma recurrence will be blinded to treatment assignment until completion of the final analysis for each cohort. Only when knowledge of the investigational product is essential for a treatment decision (e.g., planning follow-on therapy in a patient who exhibits melanoma recurrence prior to the final analysis), clinical management, or the welfare of the patient, the treating physician may request to unblind a patient's treatment assignment. In such cases, the individual patient's treatment assignment will be unblinded to the treating physician. Unblinding requires prior approval of the Clinical Principal Investigator. Local pharmacists, limited pharmacy personnel, limited laboratory personnel and personnel of the Trial coordination & Data Center (TDC) will be unblinded during the trial.

8.2 Statistical analysis

A patient will be considered to be eligible if he/she did not have any major deviations from the patient entry criteria listed in chapter 3 of the protocol. Eligibility will be assessed by the study coordinators based on the review of each patient file.

8.2.1 Analysis methods for efficacy endpoints

All the main analyses of the efficacy endpoints (RFS, OS) will be performed on the intention-to-treat (ITT) population. The ITT population is defined as all eligible, randomized patients in the arm they were allocated by randomization. The ITT principle will be followed for these analyses: all patients will be considered at risk of having an event until the respective event has been reached; the follow-up will not be censored even if patient went off-protocol treatment due to protocol violation, toxicity, treatment refusal, etc. Besides, a per protocol (PP) analysis is performed. This is defined as a subset of the ITT population who completed the study without any major protocol violations. In the PP analysis we will exclude all protocol violators, including anyone who did not adhere to treatment, switched groups, or missed crucial measurements.

The Kaplan-Meier technique will be used to obtain estimates of the time to event distributions (e.g. RFS, OS), and compare between arms using the log-rank test, stratified by stage of the disease, adjuvant radiotherapy, BRAF mutation status, HLA-type and nDC production centre as indicated at randomization. Medians will be presented with a 90% confidence interval.
The Hazard Ratio (HR), and its 90% confidence interval, of nDC vaccination to placebo, will be estimated using a Cox proportional hazards model, stratified by stage of the disease, adjuvant radiotherapy, BRAF mutation status, HLA-type and nDC production centre as indicated at randomization, with treatment as the single covariate.

8.2.2 Analysis methods for safety, tolerability and cost effectiveness endpoints

All the analyses of the safety endpoints will be performed on the safety population (all patients who have started their allocated treatment (at least started leukapheresis)).

The following analyses of AE (frequency and percentage, unless otherwise noted) will be reported by treatment arm:

- Adverse events (worst CTCAE version 4.03 grade of any event)
- Serious adverse events (worst CTCAE version 4.03 grade of any event)
- Drug-related adverse events (worst CTCAE version 4.03 grade of any event)
- Drug-related serious adverse events (worst CTCAE version 4.03 grade of any event)
- Adverse events leading to discontinuation of nDC vaccination/placebo treatment (worst CTCAE version 4.03 grade of any event)
- Immune-related adverse events (worst CTCAE version 4.03 grade of any event)

Results from the EORTC QLQ-C30 will be reported.

Results from the ICER analysis will be reported.

8.2.3 Analysis methods for immunological endpoints

All analyses will be done in patients who received nDC vaccination (arm A) and placebo (arm B). Patients who did not get at least one drug administration are excluded from the analyses. The Kaplan-Meier technique will be used to obtain estimates of the time to event distributions (e.g. RFS, OS), and compared based on immunological response (see appendix 2). Medians will be presented with a 90% confidence interval. The HR, and its 90% confidence interval, of nDC vaccination to placebo, will be estimated using a landmark analysis.

8.3 Interim analysis

One interim analysis will be performed at the time at which all patients are included. The interim analysis will include both futility and efficacy. O'Brien-Fleming boundaries will be used for the analyses. The boundaries will be based on the actual number of events observed at interim, and will be calculated using the EAST software. Results of this interim analysis will be used to decide together with the Ministry of Health if extended access will be

allowed in the Netherlands in the time period after the inclusion period and before the final analysis will be performed.

Final analysis will take place when 75 events are observed in the study. In that case, a nominal significance level of 0.005 would be applied to the first interim test of futility.

8.4 Study monitoring

After 1 year of accrual, accrual will be assessed. If accrual is less than expected, which could happen in the current era with rapid emerging treatments in melanoma, sample size will be reassessed in consultation with a statistician. The sample size alterations have to be approved by the Principal Investigators, the Sponsor and the appropriate Medical Ethical Review Board (CCMO) before being changed accordingly.

9. Quality of life assessment

9.1 Rationale

Reducing mortality and morbidity is still the most important factor in clinical research. Nevertheless, issues such as reducing side effects, symptom relief and improving patients' satisfaction have also become relevant parameters in the evaluation of medical strategies due to the increasing costs of medical care, especially in melanoma. Cancer treatments may produce AE and diminish the QoL even when survival is extended.

Patients with malignant melanoma likely have an impaired QoL, but this has not been systematically assessed in large scale randomized studies.[53] The current treatments in metastatic melanoma, including anti-CTLA-4, anti-PD-1 and BRAF-inhibitors, are currently under study in the adjuvant setting. Recently, ipilimumab (anti-CTLA-4 monoclonal antibodies) was approved by the FDA based on a benefit in RFS. Data on OS are awaiting. Ipilimumab has not been approved by the EMA (yet), due to the high toxicity it is questionable whether ipilimumab, similar to IFN-α, will become standard adjuvant treatment in high-risk stage III melanoma. These treatments, particularly anti-CTLA4, show substantial toxicity in metastatic patients and might have an unacceptable impact on QoL in the adjuvant setting.

Therefore, in this study, QoL is an important secondary endpoint. The main objective of QoL assessment within this clinical trial is to determine the impact of adjuvant nDC vaccination versus placebo on the patient's wellbeing. The hypothesis is that we expect no clinically relevant differences in QoL between nDC vaccination and placebo.

9.2 Quality of life instrument

QoL will be assessed with the EORTC QLQ-C30 (appendix 3) and the FACT-M, a melanoma-specific questionnaire (appendix 5). The EORTC QLQ-C30 questionnaire is composed of multi-item and single scales. These include five functional scales (physical, role, emotional, social, and cognitive), three symptoms (fatigue, nausea/vomiting and pain), a global health status/QoL scale and six single items (dyspnea, insomnia, appetite loss, constipation, diarrhea and financial difficulties). All scales and single items meet the standards for

reliability. The reliability and validity of the questionnaire is highly consistent across different language-cultural groups.[54] To assess the quality of the health status of the patients a validated so-called HRQoL instrument will be used, the EQ-5D (appendix 3). This HRQoL instrument will be completed by the patients. The EQ-5D is a generic HRQoL instrument comprising five domains: mobility, self-care, usual activities, pain/discomfort and anxiety/depression. The EQ-5D index is obtained by applying predetermined weights to the five domains. This index gives a societal-based global quantification of the patient's health status on a scale ranging from 0 (death) to 1 (perfect health). Patients will also be asked to rate their overall HRQoL on a visual analogue scale (EQ- 5D VAS) consisting of a vertical line ranging from 0 (worst imaginable health status) to 100 (best imaginable).

9.3 Study design

Patients are eligible for the QoL assessment in this study if they fulfill the eligibility criteria (see chapter 3), provided written informed consent and, more importantly, complete the baseline QoL questionnaire before the start of treatment. Patients will be informed in the patient informed consent form that they will have their QoL assessment regularly while involved in this trial. QoL will be a secondary outcome and evaluated in a longitudinal design for in all patients entered in this study.

QoL questionnaires must be filled out within a week before, or at the hospital, when the patient comes for a scheduled visit. If the patient did not fill the questionnaires out before the visit, they will be asked to fill it out at the hospital or, if not feasible, at home as soon as possible, but no later than 5 days after the visit.

Patients will be asked to fill out the questionnaires as completely and accurately as possible. Master copies of the QoL questionnaires (EORTC QLQ-C30; EQ-5D) will be sent to the institution. Time windows for eligible follow-up assessment will be (+/-) 3 weeks. The questionnaire must be completed within 2 weeks prior to randomization, and at month 3 till month 60 after randomization, varying from 3 to 12 months recall or until recurrence of disease.

9.4 Statistical considerations

The sample size for this trial has been calculated based on expected differences in the primary endpoint (2-year RFS rate) at 210 patients. Primary QoL outcomes that will be used to test the null hypothesis of no difference between treatment arms are global health status scores and EQ-5D scores. Based on the work of Osoba et al. a difference of 10 points on a 100 point scale between the two treatment arms will be considered as clinically significant.[55] Data will be scored according to the algorithm described in the EORTC QLQ-C30 scoring manual.

The QoL scores in the two arms will be compared by using summary statistics. Three summary statistics will be calculated per patient: the baseline score, differences from baseline to 3, 6 and 12 months, the average score reported during the treatment phase (first 1.5 years) and the average score reported after the treatment phase (3.5 years). Non-parametric rankorder tests will be performed using a two-sided significance level of 5% to test for significant differences between the treatment arms. A longitudinal repeated measures approach using all data available for a patient will be used to analyze difference in QoL between the treatment arms from baseline to stated time point. Change from baseline will be reported in a descriptive manner to provide support for the main results.

When performing a QoL analyses bias may arise due to missing data. This issue has a bearing on whether a valid comparison of the treatment arms can be made. In order to assess the impact of the missing data, sensitivity analysis will be carried out using data where missing values have been replaced using regression based imputation. Treatment arms will be compared in terms of QALY, where QALY are calculated by combining EQ-5D with survival data.

10. Economic evaluation

10.1 Clinical background

10.1.1 Rationale

Melanoma is the most deadly form of skin cancer, which mainly occurs in healthy, young working people, and has an important public health concern.

Given the substantial health burden and cost of treatment associated with melanoma, it is important to examine the economics associated with its treatment, especially with current expensive new treatment options in stage IV melanoma which are studied in the adjuvant setting. If these treatments will become standard of care in stage III melanoma, costs will increase substantially (up to €60.000 to €80.000 a year). The purpose of the economic evaluation of this study is to investigate the potential efficiency of nDC vaccination versus placebo in high-risk stage IIIB and IIIC melanoma patients after standard surgical treatment from a societal perspective. The economic evaluation is based on the general principles of a cost-effectiveness analysis and is performed along-side the randomized clinical trial.

We hypothesize to show a 20% improvement in 2-year RFS rate and diminish the proportion of patients who will develop distant metastases. In case of distant metastases current treatments are highly expensive and have substantial side effects. With reducing the risk of recurrence we hope to improve QoL and diminishing costs. Furthermore, if effectiveness of nDC vaccination is similar to currently tested drugs in stage III melanoma, anti-CTLA4, anti-PD-1 and BRAF-inhibitors, it is most likely to be beneficial in costs. Currently tested drugs in the adjuvant setting are described in table 1.

Table 1. Drugs that are currently tested in phase III melanoma in the adjuvant setting and expected additional costs compared to current standard of care (follow-up).

clinicaltrials.gov identifier	Drug tested and control arm	Expected costs after proven efficacy
NCT01274338	Ipilimumab (anti-CTLA-4) versus recombinant IFN-α	€ 80.000 per patient per year
NCT02388906	Nivolumab (anti-PD-1) versus ipilimumab (anti-CTLA-4)	€ 80.000 per patient per year
NCT02362594	Pembrolizumab (anti-PD-1) versus placebo	€ 80.000 per patient per year
NCT01682083	Dabrefenib (BRAF inhibitor) combined with Trametinib (MEK inhibitor) versus placebo	€ 80.000 per patient per year
NCT01667419	Vemurafenib (BRAF inhibitor) versus placebo	€ 60.000 per patient per year

10.1.2 Health economics literature review

Based on a recent systematic review estimated total costs of stage III melanoma patients per month are $4415, $1574 and $3567 for the initial, interim and terminal phase, based on data from various countries including the United States, Australia and European countries.[56] The interim phase corresponds with the stage IIIB and IIIC melanoma patients included in this trial, after initial surgery and before developing recurrent melanoma. Total costs of stage III melanoma patients vary from $31.778 to $68.220.

Since a systemic review regarding the cost effectiveness of DC vaccinations is not available, we discuss this based on our own experience as well as those of international experts in the field, Prof Thielemans, Brussels, Belgium and Prof Schuler, Erlangen, Germany. Expected costs of 3 cycles of nDC vaccination are around €37.500, including leukapheresis, production of nDC, administration of nDC and clinical costs.

10.2 Objective

10.2.1 Aim of economic study

Primary outcome measures for the economic evaluation, considering both the study treatment phase and follow-up period, are costs (direct and indirect), QoL, survival and the composite measure ICER.

10.2.2 Perspective

The analysis will be conducted from the viewpoint of the health care provider.

10.3 Methods

10.3.1 Type of economic evaluation

This study investigates the potential efficiency of nDC vaccination versus wait and see in high-risk stage III melanoma patients after standard treatment from a societal perspective. The economic evaluation is based on the general principles of a cost-effectiveness (utility) analysis and is performed along-side the randomized clinical trial. Besides the time frame of the trial we want to make inferences on a longer time frame, i.e., 5 years for the entire target population.

10.3.2 Choice of comparator

For the base-base empirical analysis the comparator of patients in the nDC vaccination arm (arm A) are the patients receiving placebo (arm B).

10.3.3 Patient population

See chapter 3.

10.3.4 Measurement of resource use

Costs will be measured as the sum of the costs of the intervention, hospitalization, outpatient medical care, medications during study and after recurrence of disease, and societal costs (loss of income). They will be calculated by multiplying measures of resource

use and estimates of unit costs for each of the resources. The cost analysis exists of two main parts. First, on patient level, volumes of care will be measured prospectively using medical records and the Medical Consumption Questionnaire (MCQ) (appendix 8). Resource use, such as doctor's visits, treatment (including nDC vaccinations, chemotherapy, immunotherapy, surgery, radiotherapy and targeted therapy), hospital (re)admissions, diagnostics, as well as out-of-pocket expenses such as for over-the-counter drugs and travel costs to an outpatient clinic will be collected. Where relevant and possible, (missing) cost questionnaire entries will be verified by data from the medical records. The economic evaluation will be incremental with a strong focus on the cost drivers. Productivity losses for patients will be estimated using the short Health and Labour questionnaire and will be used for economic assessment (see appendix 7) at 3 up to maximal 60 months after randomization varying from 3 to 12 months recall. The friction cost-method will be applied following the Dutch guidelines.

10.3.5 Measurement of unit costs

The second part of the cost analysis consists of determining the cost prices for each volume of resource consumption in order to use these for multiplying the volumes registered for each participating patient. The Dutch guidelines for cost analyses will be used (Hakkaart-van Roijen et al., CVZ 2010). For units of care/resources where no guideline or standard prices are available real cost prices will be determined, for example for nDC vaccination.

10.3.6 Measurement of effectiveness

To measure the quality of the health status of the patients a validated HRQoL instrument will be used, the EQ-5D. This HRQoL instrument will be completed by the patients and is available in a validated Dutch translation. The EQ-5D is a generic HRQoL instrument comprising five domains: mobility, self-care, usual activities, pain/discomfort and anxiety/depression. The EQ-5D index is obtained by applying predetermined weights to the five domains. This index gives a societal-based global quantification of the patient’s health status on a scale that is (upper) anchored at 1 (perfect health). Patients will also be asked to rate their overall HRQoL on a visual analogue scale (EQ- 5D VAS) consisting of a vertical line

ranging from 0 (worst imaginable health status) to 100 (best imaginable). QoL will be combined with survival providing in QALY.

10.3.7 Time horizon

Besides the time frame of the study we want to make inferences on a longer time frame, i.e., 5 years for the entire target population.

10.4 Statistical analysis

Statistical analysis will be done on the ITT population. For this analysis we will, additionally to the empirical economic evaluation alongside the trial, build a decision analytical discounted cost-effectiveness model that will be analyzed in a probabilistic fashion. Input for the modeling part stems largely from the study completed with information from earlier studies and secondary data.

Cumulative costs per arm will be calculated using for example the Lin method for censored cost analysis. QALY will be calculated by summing the area under each individual's QALY curve (constructed by plotting the feeling thermometer and Health Utilities Index scores for each interview during follow-up).

The empirical efficiency analysis will be analyzed using appropriate methods depending on the nature of the data. The net monetary benefit statistic will be used when there appears to be coincidental imbalance allocation in some observed covariates (baseline variables). In case of a balanced allocation of baseline variables the ICER, based on EQ-5D utilities, will be computed and uncertainty will be determined using the bootstrap method (empirical part) and 1st and 2nd order Monte Carlo simulation (modeling part). A cost-effectiveness acceptability curve will be derived that is able to evaluate efficiency by using different thresholds (willingness to pay for a QALY). The impact of uncertainty surrounding deterministic parameters (for example prices) on the ICER will be explored using one-way sensitivity analyses on the range of extremes. Secondary ICER as 'cost per recurrence prevented' and 'cost per life year gained' will also be reported, in the same fashion as described above.

Additionally, a decision analytical economic evaluation model will be made to compare costs and effects of nDC vaccination to other (upcoming) adjuvant treatments.

10.5 Reporting of results

Publication of the economic analysis will follow the "Uniform Requirements for Manuscripts Submitted to Biomedical Journals" of the International Committee of Medical Journal Editors (ICMJE). Authorship will be decided accordingly with description of the contributions of each author. In general, all submitted economic analyses will have the responsible health economist as primary author.

10.6 Collaboration with local health economics research groups

Collaboration with the Health Economics Research Group in our centre, Radboudumc, to assist with local unit cost tracking, overall cost assessment and participation in the economic study is ensured.

11. Patient registration / randomization procedure

11.1 Patient identification

Patient registration will only be accepted from authorized Investigators.

A patient can only be registered after signature of the patient informed consent. The patient is registered in the e-CRF and a patient identification number will be assigned. The registration section of the electronic CRF (e-CRF) must be completed by the responsible Investigator or its delegate. General demographic details and tumor characteristics will be entered in the screening section for each patient. At the end of the registration and assessing eligibility, a patient will be randomized.

11.2 Randomization

Randomization will be performed maximally 1 week before the first leukapheresis and only after having verified that the patient is fully eligible and having obtained the patient's informed consent to participate in the study.

Randomization of patients and treatment allocation at the Investigator site will be performed using a central randomization system. The randomization algorithm will use a minimization procedure accounting for the following stratification factors:

- Stage of the disease (IIIB versus IIIC).
- Adjuvant radiotherapy (planned/received versus not planned/not received)
- BRAF mutation status (BRAF wild type versus BRAFV600 mutation (versus unknown))
- HLA-type (HLA-A2 negative versus HLA-A2 positive)
- nDC production centre (Nijmegen versus Amsterdam)

A BRAF mutation analyses should be performed on tumor tissue of all patients. In the event when the BRAF mutation analyses cannot be performed or is inconclusive, patients will be stratified based on a unknown BRAF mutation status. A maximum of 50% may be stratified with a unknown BRAF status.

As soon as the targeted number of randomized patients has been reached, the enrolment of patients will be frozen. However, to allow patients at the screening phase or those who have

signed the informed consent form at the time of enrolment hold to be enrolled, an over-enrolment of 5% will be allowed.

11.3 Method of blinding and breaking the study blind

The study will be a double-blind trial. Blinding of the treatment is critical to the integrity of this study. Neither the treatment arm (arm A) nor the placebo arm (arm B) will be provided to the Clinical Principal Investigators, Local Investigators, the Sponsor, patients and site staff. The local pharmacists, limited pharmacy personnel, limited laboratory personnel and personnel of the TDC will be unblinded.

Only when knowledge of the investigational product is essential for a treatment decision (e.g., planning follow-on therapy in a patient who exhibits melanoma recurrence prior to the final analysis), clinical management, or the welfare of the patient, the Investigator may request to unblind a patient’s treatment assignment. Before breaking the blind of an individual patient's blinded treatment, the Investigator must have determined that the information is necessary, i.e., that it will alter the patient's immediate management. In such cases, the individual patient’s treatment assignment will be unblinded to the treating Investigator. In many cases, particularly when the emergency is clearly not investigational product-related, the problem may be properly managed by assuming that the patient is receiving active product without the need for unblinding. Unblinding requires prior approval of the Clinical Principal Investigator.

12. Form and procedures for collecting data

12.1 Case report forms and schedule for completion

Data will be collected on an e-CRF to document eligibility, safety and parameters necessary to evaluate the study endpoints. Additionally, data on the primary tumor will be collected. The e-CRF is developed by the TDC, Nijmegen.
The e-CRF will be completed on site by the local Investigator or an authorized staff member. All e-CRF entries must be based on source documents. Guidelines for completing the e-CRF and training materials will be provided by the TDC. Access to the e-CRF will be provided by the TDC only to authorized site staff members. QoL questionnaires will be entered in the e-CRF. When used, hardcopy QoL questionnaires will be archived at the sites. Further details on the collection of data are described in a datamanagementplan.

Data will be electronically reported by all participants in the e-CRF.
A. Before the treatment starts:
- The patient must be registered and randomized as described in chapter 11
B. Upon occurrence of a Pregnancy
- Any pregnancy in a female subject diagnosed during the treatment period or within 30 days after last study treatment administration must be reported to the study coordinator.
- Upon notification of a pregnancy, it will be the responsibility of the Sponsor to follow up the development and outcome of the pregnancy.
C. Upon occurrence of a SAE (see chapter 13).

12.2 Data flow

The e-CRF must be completed by the Investigator or an authorized staff member as soon as the requested information is available. The list of staff members authorized to fill in e-CRFs must be sent by the responsible Investigators to the TDC. Data collected on the e-CRF will be verified for accuracy by the central data manager. If necessary, queries will be sent to the investigational site to clarify the data on the e-CRF. The Investigator should answer data

queries within the specified time line. All original source data should be readily available for review. Modifications in the e-CRF will be tracked via an audit trail.

13. Safety reporting

13.1 Section 10 WMO event

In accordance to section 10, subsection 4, of the WMO, the sponsor will suspend the study if there is sufficient ground that continuation of the study will jeopardise subject health or safety. The sponsor will notify the Dutch Central Committee on Research Involving Human Subjects (CCMO) without undue delay of a temporary halt including the reason for such an action. The study will be suspended pending a further positive decision by the CCMO. The investigator will take care that all subjects are kept informed.

13.2 Adverse events

An **AE** is defined as any untoward medical occurrence or experience in a patient which occurs following the administration of the trial medication regardless of the dose or causal relationship. This can include any unfavorable and unintended signs (such as rash or enlarged liver), or symptoms (such as nausea or chest pain), an abnormal laboratory finding (including blood tests, x-rays or scans) or a disease temporarily associated with the use of the protocol treatment. All adverse events reported spontaneously by the subject or observed by the Investigator or his staff will be recorded in the e-CRF. Abnormal laboratory values or test results occurring after informed consent constitute AE only if they induce clinical signs or symptoms, are considered clinically significant, require therapy.

The relationship of an AE to the trial medication is determined as follows:

Almost certainly	• Starts within a time related to the study drug administration and<br>• No obvious alternative medical explanation.
Probably	• Starts within a time related to the study drug administration and<br>• Cannot be reasonably explained by known characteristics of the patient's clinical state.
Possibly	• Starts within a time related to the study drug administration and<br>• A causal relationship between the study drug and the adverse event is at least a reasonable possibility.
Unlikely	• The time association or the patient's clinical state is such that the study drug is not likely to have had an association with the observed effect.
Unrelated	• The AE is definitely not associated with the study drug administered.

13.3 Serious adverse events

A **SAE** is defined as any undesirable experience occurring to a patient, whether or not considered related to the protocol treatment which results in:

- death;
- a life threatening situation (i.e. the patient was at immediate risk of death at the time the reaction was observed);
- hospitalization or prolongation of existing hospitalization;
- persistent or significant disability/incapacity;
- a congenital anomaly/birth defect;
- any other medically important condition (i.e. important adverse reactions that are not immediately life threatening or do not result in death or hospitalization but may jeopardize the patient or may require intervention to prevent one of the other outcomes listed above).

SAE must be reported to the TDC, Nijmegen **within 24 hours** after the event was known to the Investigator as described in data management guidelines. The TDC will forward all SAE reports within 24 hours of receipt to the Clinical Principal Investigators. The Principal

Investigator will evaluate if the SAE qualifies as a suspected unexpected serious adverse reaction (SUSAR). The Principal Investigator (delegated to the TDC) will report the SAE through the web portal ToetsingOnline to the CCMO that approved the protocol, within 15 days after the Principal Investigator has first knowledge of the SAE.
SAE that result in death or are life threatening should be reported expedited. The expedited reporting will occur not later than 7 days after the responsible Investigator has first knowledge of the SAE. This is for a preliminary report with another 8 days for completion of the report.

The following events do not require to be reported as a SAE:
- relapse of the disease under study or symptoms of relapse/progression of the disease under study;
- hospitalization for diagnostic investigations (e.g., scans, endoscopy, sampling for laboratory tests, bone marrow sampling) that are not related to an AE. Hospitalization or prolonged hospitalization for a complication of such procedures remains a reportable SAE;
- prolonged hospitalization for technical, practical, or social reasons, in absence of an AE;
- hospitalization for a procedure that was planned prior to study participation (i.e. prior to registration or randomization). This should be recorded in the source documents. Prolonged hospitalization for a complication of such procedures remains a reportable SAE.

13.4 Suspected unexpected serious adverse reactions

SUSAR are AE, of which the nature, or severity, is not consistent with the applicable product information.
Unexpected adverse reactions are SUSARs if the following three conditions are met:
- the event must be serious (see section 13.3);
- there must be a certain degree of probability that the event is a harmful and an undesirable reaction to the medicinal product under investigation, regardless of the administered dose;
- the adverse reaction must be unexpected, that is to say, the nature and severity of the adverse reaction are not in agreement with the product information as recorded in: Summary of Product Characteristics for an authorised medicinal product; Investigator's Brochure for an unauthorised medicinal product.

The expedited reporting of SUSAR through the web portal ToetsingOnline is sufficient as notification to the competent authority.

13.5 Annual safety report

In addition to the expedited reporting of SUSARs, the sponsor will submit, once a year throughout the clinical trial, a safety report to the accredited CCMO. *The annual safety report may be combined with the annual progress report (see section 16.3).*

This safety report consists of:

- a list of all suspected (unexpected or expected) SAE, along with an aggregated summary table of all reported SAE, ordered by organ system, per study;
- a report concerning the safety of the subjects, consisting of a complete safety analysis and an evaluation of the balance between the efficacy and the harmfulness of the medicine under investigation.

13.5 Follow-up of adverse events

All AE will be followed until they have abated, or until a stable situation has been reached. Depending on the event, follow up may require additional tests or medical procedures as indicated, and/or referral to the general physician or a medical specialist.

14. Quality assurance

14.1 Control of data consistency

Data will be collected on e-CRF and saved in an electronic database. Consistency checks will be performed by the TDC on newly entered forms; queries will be issued in case of inconsistencies. Inconsistent forms will be kept "pending" until resolution of inconsistencies.

14.2 On-site quality control

Representatives of the TDC will be allowed to periodically review study records and compare them with source documents directly and discuss the conduct of the study with the Local Investigator, and to verify that the facilities remain acceptable.

We applied the risk classification as outlined in the document “Kwaliteitsborging mensgebonden onderzoek 2.0”, formulated by the Netherlands Federation of University Medical Centres. For this study, we have classified the risk as negligible (“verwaarloosbaar”) for the participating patients.

Table 2: Risk classification			
Severity of AE → Chance for AE ↓	Low injury	Moderate injury	Major injury
Low	Negligible risk	Negligible risk	Moderate risk
Moderate	**Negligible risk**	Moderate risk	High risk
High	Moderate risk	High risk	High risk

14.3 Audits

To ensure quality of data, study integrity and compliance with the protocol and the various applicable regulations and guidelines, the government inspectors might conduct site visits to institutions participating in the trial.

The Investigator, by accepting to participate in this protocol, agrees to co-operate fully with any quality assurance visit undertaken. The Investigator will also grant direct access to

documentation pertaining to the clinical trial (including source documents, hospital patient charts and other study files) to these authorized individuals.

The Investigator must inform the TDC immediately in case a regulatory authority inspection is scheduled. Audit reports will be kept confidential.

15. Ethical considerations

15.1 Patient protection

The responsible Investigator will ensure that this study is conducted in agreement with the Declaration of Helsinki (October 2013) and the Medical Research Involving Human Subjects Act (WMO; December 1999).

The protocol has been written, and the study will be conducted according to the International Conference on Harmonization of Technical Requirements for Registration of Pharmaceuticals for Human Use (ICH) Harmonized Tripartite Guideline on Good Clinical Practice (ICH-GCP, available online at http://www.emea.eu.int/pdfs/human/ich/013595en.pdf). The protocol must be approved by the competent ethics committee(s) as required by the applicable national legislation.

15.2 Subject identification

A sequential identification number will be automatically allocated to each patient registered in the trial. This number will identify the patient and must be included on all CRF. In order to avoid identification errors, a local patient's code (maximum of 4 letters) and year of birth will also be reported on the e-CRF.

15.3 Informed consent

When a potential participant meets the inclusion criteria (as far as known before screening procedures), the study will be explained and a letter containing information about the study, together with an informed consent form, will be presented.

The person will be asked to carefully consider this information at home, and to respond within four weeks when deciding to cooperate. Participants will be asked to sign the informed consent form.

All patients will be informed about:

- the aims of the study
- the possible AE
- the procedures and possible hazards to which the patient will be exposed

- the mechanism of treatment allocation
- strict confidentiality of any patient data
- medical records possibly being reviewed for trial purposes by authorized individuals other than their treating physician.

The informed consent documents are to be submitted to ethics committees for approval. It is the responsibility of the competent ethics committee to ensure that the translated informed documents comply with ICH-GCP guidelines and all applicable national legislation. It is emphasized in the patient information sheet that participation is voluntary and that the patient is free to refuse further participation in the protocol whenever he/she wants to. This will not have any impact on the patient's subsequent care.
Documented informed consent must be obtained for all patients included in the study before they are registered and/or randomized. The written informed consent form must be signed and personally dated by the patient or by the patient's legally acceptable representative.

16. Administrative responsibilities

16.1 Handling and storage of data and documents

The Department of Tumor Immunology, Radboudumc, will perform data management of immunological data. The TDC, Radboudumc, will perform data management of clinical and follow-up data.

Clinical and immunological data will be handled confidentially and only by the involved Investigators and people involved in the study e.g. laboratory technicians. Both clinical and immunological data are coded and linked by a unique and anonymous serial number that can be traced back to an individual patient by a local patient identification list, which is only accessible for the local authorized Investigators and local people involved in the study. Storage of study data will be in the e-CRF to which access is regulated based on the study role and site. Data and human material will be kept by serial number and stored at the Radboudumc and used for further research analysis but only for questions concerning this study.

16.2 Amendments

Amendments are changes made to the research after a favorable opinion by the CCMO has been given. A 'substantial amendment' is defined as an amendment to the terms of the CCMO application, or to the protocol or any other supporting documentation, that is likely to affect to a significant degree:

- the safety or physical or mental integrity of the subjects of the trial;
- the scientific value of the trial;
- the conduct or management of the trial; or
- the quality or safety of any intervention used in the trial.

All substantial amendments will be notified to the CCMO. Non-substantial amendments (typing errors, administrative changes etc) will not be notified to the CCMO, but will be recorded and filed.

16.3 Annual progress reports

The Investigator will submit a summary of the progress of the trial to the CCMO once a year. Information will be provided on the date of inclusion of the first subject, numbers of subjects included and numbers of subjects that have completed the study.

16.4 End of study report

The Investigator will notify the CCMO of the end of the study within a period of 90 days. The end of the study is defined as the last patient's last visit.
The sponsor will notify the CCMO immediately of a temporary halt of the study, including the reason of such an action. In case the study is ended prematurely, the sponsor will notify the CCMO and the competent authority within 15 days, including the reasons for the premature termination. Reasons for termination can be the premature termination of the temporary admission of the intervention in the basic health insurance because of insufficient inclusion, no adequate follow-up or the availability of a novel treatment modality.
Within one year after the end of the study, the Principal Investigators will submit a final study report with the results of the study, including any publications/abstracts of the study, to the CCMO.

17. Trial insurance

The Sponsor has a liability insurance which is in accordance with article 7, subsection 9 of the WMO. The Sponsor's insurance is in accordance with the legal requirements in the Netherlands (Article 7 WMO and the Measure regarding Compulsory Insurance for Clinical Research in Humans of 23th June 2003). This insurance provides cover for damage to research subjects through injury or death caused by the study:

1. € 650.000,-- (i.e. six hundred and fifty thousand Euro) for death or injury for each subject who participates in the trial;
2. € 5.000.000,-- (i.e. five million Euro) for death or injury for all subjects who participate in the trial;
3. € 7.500.000,-- (i.e. seven million five hundred thousand Euro) for the total damage incurred by the organization for all damage disclosed by scientific research for the Sponsor as 'verrichter' in the meaning of said Act in each year of insurance coverage.

The insurance applies to the damage that becomes apparent during the study or within 4 years after the end of the study.

18. Publication policy

The publication of the main trial results will be written by the study coordinators on the basis of the final analysis performed at the TDC and will be sent to a major scientific journal. The Study Coordinator and the TDC must approve all publications, abstracts and presentations of data pertaining to patients included in this study.
Authors of the manuscript will include at least the Study Coordinator, the Investigators who have included more than 5% of the eligible patients in the trial (by order of inclusion), and members of the TDC team who have contributed to the trial.
All manuscripts will include an appropriate acknowledgment section which includes:
- mentioning all Investigators who have contributed to the trial,
- the TDC staff involved in the study,
- the Netherlands Organization for Scientific Research (NWO) for financing the study
- FLIMS faculty for assistance on writing the protocol.

Authorship of any publication and/or public disclosure will be in accordance with the guidelines of the ICMJE.

Appendices

Appendix 1. Performance status WHO criteria

WHO Performance Status	
0	Able to carry out all normal activity without restriction
1	Restricted in physically strenuous activity but ambulatory and able to carry out light work
2	Ambulatory and capable of all selfcare but unable to carry out any work activities. Up and about more than 50% of waking hours
3	Capable of only limited selfcare, confined to bed or chair more than 50% of waking hours
4	Completely disabled. Cannot carry on any selfcare. Totally confined to bed or chair
5	Dead

Appendix 2. Immunomonitoring, biomarker analyses and determination of factors responsible for the variability of immune responses

Immunomonitoring

Immunomonitoring, performed throughout the protocol, will focus on the enhancement of tumor specific cytotoxic T cells in peripheral blood, upregulation of type I IFN (related) genes in peripheral blood and biopsies of DTH sites as well as dissected or biopsied tumor (if available). The collected material provides a unique opportunity to also investigate the presence of T cell subsets and their activation status (and other cell-cell interactions). Further improvement of clinical results is only to be expected by careful immunomonitoring.

Peripheral blood

Detection of responses against control antigen: The proliferative and humoral response against KLH in peripheral blood will be determined after each consecutive study drug administration. PBMC will be stimulated in vitro by the addition of KLH. Proliferation will be measured by a standard 3H-thymidine incorporation assay. Cytokines in the culture supernatant of KLH-stimulated PBMC will be analyzed by the cytokine bead assay to determine the type of immune response (T helper 1, T helper 2) induced. Pre-treatment PBMC samples will be used as controls. Serum of vaccinated patients will be used to detect anti-KLH monoclonal antibody using a standard ELISA.

Detection of type I IFN: To study the early influence of an intranodal DC vaccination (especially with pDC) on the transcriptome-signature of PBMC, peripheral blood will be drawn before and 2-4 hours after the third study drug administration in a PAXgene tube from which RNA can be isolated. RNA isolation will be performed using PAXgene blood RNA kit. mRNA levels for the type IFN-α/-β and IFN related genes will be determined by quantitative PCR.

Detection of functional T cells: The specificity of the T cells will be tested by tetramer analysis. After specificity functionality will be tested by a cytokine bead array. Also the T cells will be tested on their cytotoxic potential.

DTH biopsies

Immunohistology: Characterization of leukocyte infiltrates will be performed with antibodies against different surface markers on infiltrating mononuclear cells. The normal skin biopsy will serve as a control.

DTH-derived T cells: The T cells cultured from DTH biopsies will be stimulated and evaluated for their cytotoxic activity. The cytokine bead assay will also be used to determine cytokines secreted by T cells from the DTH cultures.

Immunologically responding patients are defined as:

Adaptive responders: T cells isolated from vaccine challenged sites (DTH biopsies) that can be expanded and 1) express T cell receptors specific for the vaccine and 2) show effector functions measured by IFN-γ secretion or cytolytic activity against tumor antigen expressing target cells.

Innate responders: positive mRNA expression of type I IFN or related genes after the third study drug administration.

Immunologically non-responding patients are defined as:

Adaptive responders: No T cells, or T cells isolated DTH biopsies that cannot be expanded, or T cells that can be expanded but do not recognize tumor antigens, or can recognize tumor antigens but do not display T effector functions i.e. lysis of tumor cell targets or release of IFN-γ.

Innate responders: no mRNA expression of type I IFN or related genes after the third study drug administration.

Biomarker analyses

The main objective of the exploratory biomarker analyses is to investigate the potential correlation of biomarkers with clinical outcome in melanoma patients, especially those treated with DC vaccination. Ideally, we will develop biomarkers that can predict which patients will benefit from DC based therapy. We have already preliminary findings from analyses of our previous DC vaccination trials, where we find that RKIP (pepb1) gene

expression in the PBMC after vaccination clearly correlates with prolonged survival. Also, immune-histological analyses of the primary tumor indicate that infiltration of the tumor with CD8+ T cells also seems to be a good prognostic factor that warrants prolonged overall survival after vaccination.

Archived material (including the primary tumor and later metastasis if available), serum, plasma and biopsies collected at baseline, during or after the trial will be used to search for biomarkers that are predictive of response to DC vaccination/immunotherapy or prognostic for melanoma. These biomarkers could be useful in future studies for the selection of patients.
The planned biomarker analyses contain the analyses of peripheral blood, DTH biopsies (see above) and T cell infiltration of the primary tumor. The definitive list of analyses may be modified when initial results from biomarker analyses become available and according to evolving knowledge of potential new markers. Tumor tissue from the primary tumor (or metastasis) will be obtained from all consenting patients. Results from the biomarker analyses will be reported separately from the main clinical study report.

Determination of factors responsible for the variability of immune responses

Genetic factors: Genetic variants affecting cytokine production in whole blood, blood mononuclear cells, and macrophages after challenge with bacterial, fungal, viral, and non-microbial stimuli will be studied. Specifically, genes that encode proteins known to influence immune responses, like the TLR1-6-10 cluster, cytokine and complement inhibitors, and the kallicrein system are of interest.

Microbiome factors: To identify associations between the gut microbiome and stimulus-induced cytokine responses, collected stool samples and whole blood will be analyzed. Fecal shotgun metagenomic sequencing will be performed to generate microbial taxonomic and functional profiles, providing a detailed characterization of the microbial community composition at the patient level as well as their functional potential. Cytokine responses will be measured ex vivo in peripheral blood mononuclear cells (PBMCs) and whole blood under five different microbial stimulations. Three stimulations will be bacteria-derived (purified

Escherichia coli-derived LPS, Bacteroides fragilis representing Gram-negative bacteria, and Staphylococcus aureus representing Gram-positive bacteria) and two will be fungal-derived, representing different morphological forms of the fungus (Candida albicans: filamentous hypha and conidia yeast). Although these stimulatory factors are pathogens, we will also include the common gut commensal organism Bacteroides fragilis, as this species can mediate development of the host immune system. TNFα and IFNγ production capacity will be studied in detail since it has appeared to be strongly influenced by the microbiome, whereas other cytokines such as IL-1β, IL-6, and Th17-derived IL-17 and IL-22 exhibit fewer, but more specific, associations with the gut microbiota.

Appendix 3. Dutch version of the EQ-5D

Gezondheidsvragenlijst
(Nederlandse versie)

Vragenlijst t.b.v. studie met adjuvante natuurlijke dendritische celvaccinatie voor stadium III melanoom patiënten

Zet bij iedere groep in de lijst hieronder een kruisje in het hokje achter de zin die het best past bij uw eigen gezondheidstoestand **vandaag**.

Mobiliteit

Ik heb geen problemen met lopen ❑
Ik heb enige problemen met lopen ❑
Ik ben bedlegerig ❑

Zelfzorg

Ik heb geen problemen om mijzelf te wassen of aan te kleden ❑
Ik heb enige problemen om mijzelf te wassen of aan te kleden ❑
Ik ben niet in staat mijzelf te wassen of aan te kleden ❑

Dagelijkse activiteiten *(bijv. werk, studie, huishouden, gezins- en vrijetijdsactiviteiten)*

Ik heb geen problemen met mijn dagelijkse activiteiten ❑
Ik heb enige problemen met mijn dagelijkse activiteiten ❑
Ik ben niet in staat mijn dagelijkse activiteiten uit te voeren ❑

Pijn/klachten

Ik heb geen pijn of andere klachten ❑
Ik heb matige pijn of andere klachten ❑
Ik heb zeer ernstige pijn of andere klachten ❑

Stemming

Ik ben niet angstig of somber ❑
Ik ben matig angstig of somber ❑
Ik ben erg angstig of somber ❑

Best voorstelbare

gezondheidstoestand

Om mensen te helpen bij het aangeven hoe goed of hoe slecht een gezondheidstoestand is, hebben we een meetschaal (te vergelijken met een thermometer) gemaakt. Op de meetschaal hiernaast betekent "100" de beste gezondheidstoestand die u zich kunt voorstellen, en "0" de slechtste gezondheidstoestand die u zich kunt voorstellen.

We willen u vragen op deze meetschaal aan te geven hoe goed of hoe slecht volgens u uw eigen gezondheidstoestand vandaag is. Trek een lijn van het hokje hieronder naar het punt op de meetschaal dat volgens u aangeeft hoe goed of hoe slecht uw gezondheidstoestand vandaag is.

Uw gezondheidstoestand vandaag

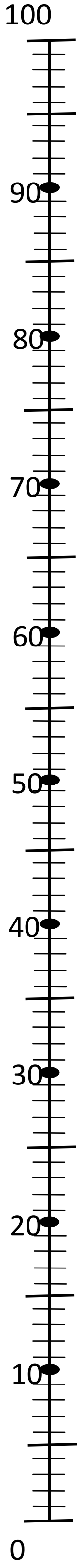

Slechtst voorstelbare

gezondheidstoestand

Appendix 4. Dutch version of the EORTC-QLQ-C30

Vragenlijst t.b.v. studie met adjuvante natuurlijke dendritische celvaccinatie voor stadium III melanoom patiënten

EORTC QLQ-C30

Wij zijn geïnteresseerd in bepaalde dingen over u en uw gezondheid. Wilt u alle vragen zelf beantwoorden door het getal te omcirkelen dat het meest op u van toepassing is. Er zijn geen "juiste" of "onjuiste" antwoorden. De informatie die u geeft wordt strikt vertrouwelijk behandeld.

1 = helemaal niet	2 = een beetje	3 = nogal	4 = heel erg

1.	Heeft u moeite met het doen van inspannende activiteiten zoals het dragen van een zware boodschappentas of een koffer?	1	2	3	4
2.	Heeft u moeite met het maken van een lange wandeling?	1	2	3	4
3.	Heeft u moeite met het maken van een korte wandeling buitenshuis?	1	2	3	4
4.	Moet u overdag in bed of in een stoel blijven?	1	2	3	4
5.	Heeft u hulp nodig met eten, aankleden, u zelf wassen of naar het toilet gaan?	1	2	3	4

1 = helemaal niet	2 = een beetje	3 = nogal	4 = heel erg

Gedurende de afgelopen week:

6.	Was u beperkt bij het doen van uw werk of andere dagelijkse bezigheden?	1	2	3	4
7.	Was u beperkt in het uitoefenen van uw hobby's of bij andere bezigheden die u in uw vrije tijd doet?	1	2	3	4
8.	Was u kortademig?	1	2	3	4
9.	Heeft u pijn gehad?	1	2	3	4
10.	Had u behoefte te rusten?	1	2	3	4
11.	Heeft u moeite met slapen gehad?	1	2	3	4
12.	Heeft u zich slap gevoeld?	1	2	3	4
13.	Heeft u gebrek aan eetlust gehad?	1	2	3	4
14.	Heeft u zich misselijk gevoeld?	1	2	3	4
15.	Heeft u overgegeven?	1	2	3	4
16.	Had u last van obstipatie? (Was u verstopt?)	1	2	3	4
17.	Had u diarree?	1	2	3	4
18.	Was u moe?	1	2	3	4

1 = helemaal niet	2 = een beetje	3 = nogal	4 = heel erg

Gedurende de afgelopen week:

19.	Heeft pijn u gehinderd in uw dagelijkse bezigheden?	1	2	3	4
20.	Heeft u moeite gehad met het concentreren op dingen, zoals een krant lezen of televisie kijken?	1	2	3	4
21.	Voelde u zich gespannen?	1	2	3	4
22.	Maakte u zich zorgen?	1	2	3	4
23.	Voelde u zich prikkelbaar?	1	2	3	4
24.	Voelde u zich neerslachtig?	1	2	3	4
25.	Heeft u moeite gehad met het herinneren van dingen?	1	2	3	4
26.	Heeft uw lichamelijke toestand of medische behandeling uw familieleven in de weg gestaan?	1	2	3	4
27.	Heeft uw lichamelijke toestand of medische behandeling u belemmerd in uw sociale bezigheden?	1	2	3	4
28.	Heeft uw lichamelijke toestand of medische behandeling financiële moeilijkheden met zich meegebracht?	1	2	3	4

Wilt u voor de volgende vragen het getal tussen 1 en 7 omcirkelen dat het meest op u van toepassing is:

29. Hoe zou u uw algehele gezondheid gedurende de afgelopen week beoordelen?

1	2	3	4	5	6	7
**Erg slecht**						**Uitstekend**

30. Hoe zou u uw algehele "kwaliteit van leven" gedurende de afgelopen week beoordelen?

1	2	3	4	5	6	7
**Erg slecht**						**Uitstekend**

Appendix 5. Dutch version of the FACT-M

Vragenlijst t.b.v. studie met adjuvante natuurlijke dendritische celvaccinatie voor stadium III melanoom patiënten

Hieronder vindt u een lijst met uitspraken die andere mensen, met dezelfde ziekte als u, belangrijk vonden. Omcirkel a.u.b. één cijfer per regel aan om uw antwoord zoals het **tijdens de afgelopen 7 dagen** van toepassing was aan te geven.

0 = helemaal niet	1 = een beetje	2 = enigszins	3 = in vrij hoge mate	4 = in zeer hoge mate

Lichamelijk welbevinden:

1.	Het ontbreekt me aan energie	0	1	2	3	4
2.	Ik voel me misselijk	0	1	2	3	4
3.	Door mijn lichamelijk toestand heb ik moeite om aan de behoeften van mijn gezin/familie tegemoet te komen	0	1	2	3	4
4.	Ik heb pijn	0	1	2	3	4
5.	Ik heb last van de bijwerkingen van de behandeling	0	1	2	3	4
6.	Ik voel me ziek	0	1	2	3	4
7.	Ik moet in bed blijven	0	1	2	3	4

Sociaal welzijn en welzijn van het gezin/familie:

8.	Ik heb een hechte band met mijn vrienden	0	1	2	3	4
9.	Ik krijg morele steun van mijn gezin/familie	0	1	2	3	4
10.	Ik krijg steun van mijn vrienden	0	1	2	3	4
11.	Mijn gezin/familie aanvaardt mijn ziekte	0	1	2	3	4
12.	Ik ben tevreden met de communicatie over mijn ziekte in mijn gezin/familie	0	1	2	3	4

0 = helemaal niet	1 = een beetje	2 = enigszins	3 = in vrij hoge mate	4 = in zeer hoge mate

13.	Ik heb een hechte relatie met mijn partner (of de persoon die mijn belangrijkste steun is)	0	1	2	3	4

Beantwoord a.u.b. de volgende vraag ongeacht het huidige niveau van uw seksuele activiteit. Als u hier liever niet wilt antwoorden, kruis dan a.u.b. dit vakje ☐ aan en ga door met het volgende gedeelte.

14.	Ik ben tevreden met mijn seksleven	0	1	2	3	4

Emotioneel welzijn:

15.	Ik voel me verdrietig	0	1	2	3	4
16.	Ik ben tevreden over hoe ik met mijn ziekte omga	0	1	2	3	4
17.	Ik verlies de hoop in de strijd tegen mijn ziekte	0	1	2	3	4
18.	Ik voel me nerveus	0	1	2	3	4
19.	Ik maak me zorgen dat ik zou kunnen doodgaan	0	1	2	3	4
20.	Ik maak me zorgen dat mijn toestand zal verergeren	0	1	2	3	4

Functioneel welbevinden:

21.	Ik ben in staat te werken (werk thuis inbegrepen)	0	1	2	3	4
22.	Mijn werk (werk thuis inbegrepen) schenkt mij voldoening	0	1	2	3	4
23.	Ik kan van het leven genieten	0	1	2	3	4

0 = helemaal niet	1 = een beetje	2 = enigszins	3 = in vrij hoge mate	4 = in zeer hoge mate

24.	Ik aanvaard mijn ziekte	0	1	2	3	4
25.	Ik slaap goed	0	1	2	3	4
26.	Ik geniet van wat ik in mijn vrije tijd doe	0	1	2	3	4
27.	Op dit moment ben ik tevreden met de kwaliteit van mijn leven	0	1	2	3	4

Bijkomende zorgen:

28.	Ik heb pijn op de plek van mijn melanoom of op de operatieplek	0	1	2	3	4
29.	Ik heb nieuwe veranderingen in mijn huid opgemerkt	0	1	2	3	4
30.	Ik maak me zorgen over hoe de littekens er na de operatie uitzien	0	1	2	3	4
31.	Ik heb last van kortademigheid	0	1	2	3	4
32.	Ik moet mijn lichaamsactiviteiten beperken vanwege mijn toestand	0	1	2	3	4
33.	Ik heb af en toe hoofdpijn	0	1	2	3	4
34.	Ik heb koortsaanvallen gehad	0	1	2	3	4

0 = helemaal niet	1 = een beetje	2 = enigszins	3 = in vrij hoge mate	4 = in zeer hoge mate

35.	Mijn maagstreek voelt gezwollen of verkramt aan	0	1	2	3	4
36.	Ik heb een goede eetlust	0	1	2	3	4
37.	Ik heb pijn in mijn botten	0	1	2	3	4
38.	Ik heb bloed in mijn stoelgang opgemerkt	0	1	2	3	4
39.	Ik moet vanwege mijn toestand mijn deelname aan sociale activiteiten beperken	0	1	2	3	4
40.	Ik voel me machteloos door mijn toestand	0	1	2	3	4
41.	Ik zonder me af van anderen vanwege mijn toestand	0	1	2	3	4
42.	Ik heb moeite om helder te denken (dingen herinneren, me concentreren)	0	1	2	3	4
43.	Ik heb last van vermoeidheid	0	1	2	3	4

Op de plek van mijn melanoomoperatie:

44.	Is de plek van mijn melanoom gezwollen	0	1	2	3	4
45.	Ben ik gezwollen als gevolg van de operatie	0	1	2	3	4
46.	Heb ik last van de hoeveelheid zwelling	0	1	2	3	4
47.	Doet het pijn als ik het gezwollen gebied beweeg	0	1	2	3	4

0 = helemaal niet	1 = een beetje	2 = enigszins	3 = in vrij hoge mate	4 = in zeer hoge mate

48.	Belet de zwelling mij de dingen te doen die ik wil doen	0	1	2	3	4
49.	Belet de zwelling mij kleren of schoenen te dragen die ik wil dragen	0	1	2	3	4
50.	Voelt mijn operatieplek gevoelloos aan	0	1	2	3	4
51.	Heb ik een goed bewegingsbereik in mijn arm of been	0	1	2	3	4

Appendix 6. Dutch version of QoL-MIND-DC questionnaire

Vragenlijst t.b.v. studie met adjuvante natuurlijke dendritische celvaccinatie voor stadium III melanoom patiënten (studiespecifieke vragen)

Omcirkel a.u.b. één cijfer per regel aan om uw antwoord aan te geven.

0 = helemaal niet	1 = een beetje	2 = enigszins	3 = in vrij hoge mate	4 = in zeer hoge mate

1.	Ik heb last gehad van bijwerkingen in de studie	0	1	2	3	4
2.	Ik heb last gehad van griepverschijnselen kort na de vaccinatie	0	1	2	3	4
3.	Ik heb last gehad van de injectieplaats	0	1	2	3	4
4.	De bijwerkingen heb ik als vervelend ervaren	0	1	2	3	4
5.	De hoeveelheid ziekenhuisbezoeken heb ik als vervelend ervaren	0	1	2	3	4
6	De bijwerkingen hebben mij doen overwegen te stoppen met de studie	0	1	2	3	4
7.	Deelname aan deze studie heeft een negatieve invloed op mijn kwaliteit van leven	0	1	2	3	4

Appendix 7. Short Health and labour questionnaire

Deze vragenlijst gaat over de gevolgen van gezondheidsproblemen voor betaald werk en onbetaald werk (bijv. huishoudelijk werk). Deze vragen hebben steeds betrekking op ***de afgelopen maand***. Met gezondheidsproblemen worden zowel uw lichamelijke als uw emotionele problemen bedoeld.

1. Hebt u momenteel betaald werk?

☐ Nee, (gaat u door naar vraag 9)

☐ Ja;

Voor hoeveel uur per week hebt u een aanstelling? ……………..… uren per week

Over hoeveel dagen zijn deze uren verdeeld? ……………… dagen

Wat is uw beroep? ………………………..……………………………………….………………….……

2. Hebt u de afgelopen maand verzuimd van betaald werk vanwege gezondheidsproblemen?

☐ Nee

☐ Ja, ik heb ……………… werkdagen verzuimd

(Ga uit van 5 werkdagen per week)

Hebt u ***langer*** dan de gehele afgelopen maand verzuimd van betaald werk vanwege gezondheidsproblemen?

☐ Nee

☐ Ja, ik heb mij ziek gemeld sinds .... *(gaat u door naar vraag 8)*

Mensen met gezondheidsproblemen moeten daarvoor soms verzuimen van hun werk.

Het kan echter voorkomen dat iemand ***wel*** op zijn werk aanwezig is, maar zijn werk minder goed doet vanwege die gezondheidsproblemen. Daarover gaan de vragen 3 t/m 7.

3. Werd u in de afgelopen maand bij uw betaald werk gehinderd door gezondheidsproblemen?

☐ Nee, in het geheel niet (gaat u door naar vraag 8)

☐ Ja, een beetje

☐ Ja, heel erg

4. Hoeveel dagen in de afgelopen maand hebt u wèl betaald werk verricht, terwijl u last had van gezondheidsproblemen?

.... dagen (De dagen waarop u helemaal niet hebt gewerkt omdat u zich ziek hebt gemeld hoeft u **niet** mee te rekenen.)

5. Wilt u aangeven hoe goed u hebt gewerkt op de dagen dat u wèl op uw werk was terwijl u last had van gezondheidsproblemen.

(Een 1 betekent dat u zeer slecht in staat was uw werk uit te voeren en een 10 betekent dat uw werk niet werd beïnvloed.)

1	2	3	4	5	6	7	8	9	10
☐	☐	☐	☐	☐	☐	☐	☐	☐	☐
zeer slecht								even goed als normaal	

6. Nu volgt een aantal uitspraken die voor mensen met gezondheidsproblemen van toepassing kunnen zijn met betrekking tot betaald werk. Geef aan hoe vaak elke uitspraak op u van toepassing was in de afgelopen maand.

Ik was wel op mijn werk, maar als gevolg van gezondheidsproblemen ....

	bijna nooit	soms	vaak	bijna altijd
had ik concentratiestoornissen	☐	☐	☐	☐
moest ik in een langzamer tempo werken	☐	☐	☐	☐
moest ik mij afzonderen	☐	☐	☐	☐
had ik meer problemen om beslissingen te nemen	☐	☐	☐	☐
moest ik werk uitstellen	☐	☐	☐	☐
moest ik werk laten overnemen door anderen	☐	☐	☐	☐
had ik andere problemen, nl.:				
....	☐	☐	☐	☐

7. Als u het werk zou moeten inhalen dat u in de afgelopen maand niet hebt kunnen verrichten vanwege uw gezondheidsproblemen, hoeveel uur zou u dan moeten werken?

.... uur (De dagen waarop u helemaal niet hebt gewerkt omdat u zich ziek hebt gemeld hoeft u **niet** mee te rekenen.)

8. Wat is uw eigen netto inkomen uit betaald werk?

(Het gaat om het bedrag dat u 'schoon' in uw handen krijgt. Voor alle duidelijkheid: het gaat alleen om uw eigen inkomen, dus zonder dat van uw eventuele partner.)

☐ € .... per week

☐ € .... per 4 weken

☐ €.. per maand

☐ € ....per jaar

☐ Weet ik niet / wil ik niet zeggen

9. Welke van de volgende situaties is op u van toepassing? Indien meerdere situaties van toepassing zijn, wilt u dan aangeven welke situatie het ***meest*** op u van toepassing is?

☐ Ik heb een betaalde baan

☐ Ik zorg voor het huishouden (en eventueel kinderen)

☐ Ik ben gepensioneerd of met prepensioen

☐ Ik ben scholier of student

☐ Ik kan (gedeeltelijk) geen betaald werk doen vanwege gezondheidsproblemen

en ben voor ....% arbeidsgeschikt

☐ Ik doe geen betaald werk om andere redenen

(Bijv. vanwege onvrijwillige werkloosheid of vrijwilligerswerk.)

10. Hebt u de afgelopen maand de volgende activiteiten verricht en hebben gezondheidsproblemen daarbij een rol gespeeld?

a. Huishoudelijk werk (bijv. eten klaar maken, huis schoonmaken, kleren wassen.)

☐ Wel gedaan, niet gehinderd door gezondheidsproblemen

☐ Wel gedaan, wel gehinderd door gezondheidsproblemen

☐ Niet gedaan, vanwege gezondheidsproblemen

☐ Niet gedaan, om andere redenen dan gezondheidsproblemen

b. Boodschappen doen (bijv. dagelijkse boodschappen, winkelen, bezoek aan bank of postkantoor.)

☐ Wel gedaan, niet gehinderd door gezondheidsproblemen

☐ Wel gedaan, wel gehinderd door gezondheidsproblemen

☐ Niet gedaan, vanwege gezondheidsproblemen

☐ Niet gedaan, om andere redenen dan gezondheidsproblemen

c. Klussen en karweitjes (bijv. onderhoud aan huis, tuin of vervoermiddelen.)

☐ Wel gedaan, niet gehinderd door gezondheidsproblemen

☐ Wel gedaan, wel gehinderd door gezondheidsproblemen

☐ Niet gedaan, vanwege gezondheidsproblemen

☐ Niet gedaan, om andere redenen dan gezondheidsproblemen

d. Dingen speciaal voor of met uw eigen inwonende kinderen (bijv. verzorging, spelen, kinderen naar school brengen, helpen met huiswerk.)

☐ Wel gedaan, niet gehinderd door gezondheidsproblemen

☐ Wel gedaan, wel gehinderd door gezondheidsproblemen

☐ Niet gedaan, vanwege gezondheidsproblemen

☐ Niet gedaan, om andere redenen dan gezondheidsproblemen

☐ Niet van toepassing

11. Hebben anderen in de afgelopen maand huishoudelijke taken die u normaal wel doet overgenomen in verband met gezondheidsproblemen?

☐ Nee

☐ Ja, namelijk (meerdere antwoorden mogelijk):

• gezinsleden voor .... uur

• andere onbetaalde mensen voor .... uur

• thuiszorg voor .... uur

• andere betaalde hulp voor .... uur

………

Algemene vragen:

Wat is de hoogst genoten opleiding die u ***met een diploma*** hebt afgesloten?

☐ Basisschool (lager onderwijs, speciaal onderwijs)

☐ Lager beroepsonderwijs (bijv. LTS, LHNO, LEAO, huishoudschool)

☐ Middelbaar algemeen onderwijs (bijv. VMBO, ULO, MULO, MAVO)

☐ Middelbaar beroepsonderwijs (bijv. MTS, MEAO, MHNO)

☐ Voortgezet algemeen onderwijs (bijv. HBS, MMS, HAVO, VWO, gymnasium)

☐ Hoger beroeps onderwijs (bijv. HTS, HEAO, HHNO)

☐ Wetenschappelijk onderwijs

☐ Anders, namelijk:

………

2. Wat is op dit moment uw burgerlijke staat?

☐ Alleenstaand

☐ Gehuwd/duurzaam samenwonend

☐ Gescheiden

☐ Weduwe(naar)

Wij danken u voor de tijd en moeite die u genomen hebt om deze lijst in te vullen.

Hebt u nog op- of aanmerkingen of aanvullingen, dan horen wij dat graag:

……

……

……

Appendix 8 Dutch version of the iMTA Medical Consumption Questionnaire

Vragenlijst t.b.v. studie met adjuvante natuurlijke dendritische celvaccinatie voor stadium III melanoom patiënten

Bron: C. Bouwmans, L. Hakkaart-van Roijen, M. Koopmanschap, M. Krol, H. Severens, W. Brouwer. Handleiding *i*MTA Medical Cost Questionnaire (*i*MCQ). Rotterdam: iMTA, Erasmus Universiteit Rotterdam, 2013)

Deze vragenlijst gaat over uw zorggebruik in de **afgelopen 3 maanden**.

Wat is de hoogste opleiding die u heeft afgemaakt?
Zoek uw hoogste opleiding en kruis het hokje daarvoor aan.

- ☐ Ik heb geen school of opleiding afgemaakt
- ☐ Lagere school of basisschool
- ☐ Huishoudschool, vbo, lbo, lts, leao of lhno
- ☐ Mavo, mulo, ivo of vmbo
- ☐ Mbo, mts, meao, mhno, inas of intas
- ☐ Havo, vwo, hbs, mms, atheneum of gymnasium
- ☐ Hbo, hts, heao of hhno
- ☐ Universiteit
- ☐ Ik heb een andere opleiding afgemaakt, namelijk …………
  …………………………………………………………………….

Wat doet u in het dagelijks leven?
Kruis aan wat u de meeste tijd doet.

- ☐ Ik zit op school, ik studeer
- ☐ Ik werk in loondienst
- ☐ Ik ben zelfstandig ondernemer
- ☐ Ik ben huisvrouw, huisman
- ☐ Ik ben werkloos
- ☐ Ik ben arbeidsongeschikt, voor … %
- ☐ Ik ben met pensioen of prepensioen
- ☐ Ik doe iets anders, namelijk ………………………………………………….
  ………………………………………………………………………

Wij willen graag weten met welke dokters u in de afgelopen 3 maanden een afspraak had. Het gaat om afspraken voor uzelf. Ook andere zorgverleners tellen mee. Bijvoorbeeld de fysiotherapeut.
Welke afspraken tellen mee?

- Controles
- Afspraken omdat u een lichamelijke of psychische klacht had
- Afspraken waarbij de dokter bij u thuis kwam
- Telefonische afspraken
- Telefoontjes met de receptenlijn

Wat telt niet mee?

- Afspraken voor een ander, bijvoorbeeld voor uw kind
- Telefoontjes om een afspraak te maken

Weet u niet precies hoeveel afspraken het waren? Schrijf dan op hoeveel het er ongeveer waren.

1. Hoeveel afspraken had u in de afgelopen 3 maanden met uw huisarts?
- ☐ Geen enkele afspraak
- ☐ ...... afspraken

2. Hoeveel afspraken had u in de afgelopen 3 maanden met een maatschappelijk werker?
- ☐ Geen enkele afspraak
- ☐ …… afspraken

3. Hoeveel afspraken had u in de afgelopen 3 maanden met een fysiotherapeut? Of met een caesartherapeut, therapeut mensendieck of een manueel therapeut? Tel alle afspraken met deze therapeuten bij elkaar op.
- ☐ Geen enkele afspraak
- ☐ …… afspraken

4. Hoeveel afspraken had u in de afgelopen 3 maanden met een ergotherapeut?
- ☐ Geen enkele afspraak
- ☐ …… afspraken

5. Hoeveel afspraken had u in de afgelopen 3 maanden met een logopedist?
- ☐ Geen enkele afspraak
- ☐ …… afspraken

6. Hoeveel afspraken had u in de afgelopen 3 maanden met een diëtist?
- ☐ Geen enkele afspraak
- ☐ …… afspraken

7. Hoeveel afspraken had u in de afgelopen 3 maanden met een homeopaat? Of met een acupuncturist? Tel alle afspraken met deze zorgverleners bij elkaar op.
- ☐ Geen enkele afspraak
- ☐ …… afspraken

8. Hoeveel afspraken had u in de afgelopen 3 maanden met een psycholoog? Of met een psychotherapeut of psychiater? Tel alle afspraken met deze zorgverleners bij elkaar op.
- ☐ Geen enkele afspraak
- ☐ …… afspraken

9. Hoeveel afspraken had u in de afgelopen 3 maanden met de bedrijfsarts?
- ☐ Geen enkele afspraak
- ☐ …… afspraken

10. Heeft u in de afgelopen 3 maanden hulp van de thuiszorg gehad?
- ☐ Nee
- ☐ Ja

Heeft u "Ja" aangekruist? Beantwoord dan vraag 11 tot en met 13. Ga anders verder met vraag 14.

11. Wat voor hulp van de thuiszorg heeft u gehad in de afgelopen 3 maanden? U kunt meer dan 1 hokje aankruisen.

- ☐ Huishoudelijke hulp
  voorbeeld: stofzuigen, bed opmaken, boodschappen doen
- ☐ Verzorging van uzelf
  voorbeeld: hulp bij douchen of aankleden
- ☐ Verpleging
  voorbeeld: verband omdoen, medicijnen geven, bloeddruk meten

12. Hoeveel weken heeft u deze thuiszorg gehad? Tel alle weken in de afgelopen 3 maanden bij elkaar op. *Let op: een periode van 3 maanden telt 13 weken*.

- Huishoudelijke hulp: … weken in de afgelopen 3 maanden
- Verzorging van uzelf: … weken in de afgelopen 3 maanden
- Verpleging: … weken in de afgelopen 3 maanden

13. Hoeveel uur thuiszorg kreeg u in deze weken gemiddeld?

- Huishoudelijke hulp: gemiddeld … uur in de week
- Verzorging van uzelf: gemiddeld … uur in de week
- Verpleging: gemiddeld … uur in de week

14. Heeft u in de afgelopen 3 maanden medicijnen gebruikt?

- ☐ Ja
- ☐ Nee

Heeft u 'ja' aangekruist? Vul dan bij vraag 15 in welke medicijnen en hoeveel. Ga anders verder met vraag 16.

15. Welke medicijnen heeft u in de afgelopen 3 maanden gebruikt? Met medicijnen bedoelen we alle medicijnen die u hebt gekregen op recept en geneesmiddelen die u hebt gekocht bij de apotheek of de drogist. U ziet eerst drie voorbeelden.

Let op: pak de verpakking erbij! Daarop staat hoeveel u per keer moest nemen. En hoe vaak u dat moest doen. Heeft u meer of minder gebruikt? Vul dan in hoeveel u ook echt gebruikt heeft.

Hoe heet het medicijn?	Hoeveel heeft u per keer genomen?<br>Kijk op de verpakking	Hoe vaak op een dag heeft u dit gedaan?<br>Kijk op de verpakking	Op hoeveel dagen in de afgelopen 3 maanden heeft u het medicijn gebruikt?
voorbeeld 1<br>Metoprolol (tegen hoge bloeddruk)	*voorbeeld*<br>100mg	*voorbeeld*<br>1 keer	*voorbeeld*<br>90 dagen
voorbeeld 2<br>Hydrocortison crème	*voorbeeld*<br>-	*voorbeeld*<br>1	*voorbeeld*<br>14 dagen
……………………………	……………………………	……………………………	……………………………

16. Hoe vaak bent u in de afgelopen 3 maanden op de spoedeisende eerste hulp van een ziekenhuis geweest? Een andere naam voor spoedeisende eerste hulp is EHBO.
- ☐ Geen enkele keer
- ☐ … keer

17. Hoe vaak bent u in de afgelopen 3 maanden met een ambulance naar het ziekenhuis gebracht? Een andere naam voor ambulance is ziekenauto
- ☐ Geen enkele keer
- ☐ … keer

18. Had u in de afgelopen 3 maanden een afspraak bij de polikliniek van het ziekenhuis? Het gaat om afspraken voor uzelf met een dokter. Bijvoorbeeld met de cardioloog, reumatoloog of neuroloog.
- ☐ Nee
- ☐ Ja

Heeft u "Ja" aangekruist? Vul dan bij vraag 19 in bij welke soorten dokters u geweest bent. En hoe vaak. Op de eerste rij staat een voorbeeld. Ga anders verder met vraag 20.

19. Bij welke soorten dokters bent u in de afgelopen 3 maanden in het ziekenhuis geweest? En hoe vaak?

Bij welke soort dokter bent u in het ziekenhuis geweest?	*Hoe vaak bent u in de afgelopen 3 maanden bij deze dokter geweest?*
voorbeeld<br>cardioloog	*voorbeeld*<br>2 keer
……………………………	……………………………
……………………………	……………………………

20. Bent u in de afgelopen 3 maanden overdag in het ziekenhuis geweest voor een behandeling? U bleef dus niet slapen. U kwam bijvoorbeeld voor een bloedtransfusie, nierdialyse of chemokuur. De aferese en de vaccinaties tellen niet mee.

- ☐ Nee
- ☐ Ja

Heeft u "Ja" aangekruist? Beantwoord dan vraag 21 en 22.
Ga anders verder met vraag 23.

21. Voor welke soort behandeling was dit? Was dit voor meer dan 1 soort behandeling? Vul dan alle soorten behandelingen in.

- Behandeling 1: ………………………………………………………………………
- Behandeling 2: ………………………………………………………………………
- Behandeling 3: ………………………………………………………………………

22. Hoeveel keer moest u in de afgelopen 3 maanden voor deze behandelingen naar het ziekenhuis?

- ...... keer voor behandeling 1
- ...... keer voor behandeling 2
- ...... keer voor behandeling 3

23. Bent u in de afgelopen 3 maanden ergens anders geweest voor een behandeling overdag? U bleef dus niet slapen. U ging bijvoorbeeld naar de dagopvang van een woon-/zorgcentrum of een psychiatrische instelling. Of naar de dagbehandeling van een revalidatiecentrum.

- ☐ Nee
- ☐ Ja

Heeft u "Ja" aangekruist? Beantwoord dan vraag 24 en 25. Ga anders verder met vraag 26.

24. Wat voor instelling was dit? Kruis het goede antwoord aan. U kunt meer dan 1 hokje aankruisen.
- ☐ Woon-/zorgcentrum
- ☐ Revalidatiecentrum
- ☐ Psychiatrische instelling
- ☐ Een andere instelling, namelijk ………………………………………………………………………………

25. Hoe vaak moest u hier in de afgelopen 3 maanden naartoe? Heeft u bij vraag 24 meer dan 1 hokje aangekruist? Vul dan hieronder voor iedere instelling in hoe vaak u er bent geweest.
- Naar het woon-/zorgcentrum: …… keer in de afgelopen 3 maanden
- Naar het revalidatiecentrum: …… keer in de afgelopen 3 maanden
- Naar de psychiatrische instelling: …… keer in de afgelopen 3 maanden
- Naar de andere instelling: …… keer in de afgelopen 3 maanden

26. Heeft u in de afgelopen 3 maanden weleens in het ziekenhuis gelegen? U moest dus blijven slapen. Bijvoorbeeld omdat u geopereerd was en niet meteen naar huis kon.
- ☐ Nee
- ☐ Ja

Heeft u "Ja" aangekruist? Beantwoord dan vraag 27 en 28. Ga anders verder met vraag 29.

27. Hoe vaak heeft u in de afgelopen 3 maanden in het ziekenhuis gelegen?
…… keer in de afgelopen 3 maanden

28. Hoe lang heeft u in het ziekenhuis gelegen? Heeft u meer dan 1 keer in het ziekenhuis gelegen in de afgelopen 3 maanden? Tel dan alle dagen bij elkaar op.
…… dagen in totaal in de afgelopen 3 maanden

29. Moest u in de afgelopen 3 maanden ergens anders blijven slapen voor uw gezondheid? Bijvoorbeeld in een woon-/zorgcentrum, psychiatrische instelling of revalidatiecentrum.
- ☐ Nee
- ☐ Ja

Heeft u "Ja" aangekruist? Beantwoord dan vraag 30 en 31. Ga anders naar het einde van de vragenlijst.

30. Wat voor instelling was dit? U kunt meer dan 1 hokje aankruisen.
- ☐ Woon-/zorgcentrum
- ☐ Revalidatiecentrum
- ☐ Psychiatrische instelling
- ☐ Een andere instelling, namelijk ………………………………………………………………………………

31. Hoe lang bent u in deze instelling geweest? Heeft u bij vraag 30 meer dan 1 hokje aangekruist? Vul dan hieronder voor iedere instelling in hoe lang u er bent geweest. Bent u ergens meer dan 1 keer geweest in de afgelopen 3 maanden? Tel dan alle dagen bij elkaar op.

- In het woon-/zorgcentrum: …… dagen in de afgelopen 3 maanden
- In het revalidatiecentrum: …… dagen in de afgelopen 3 maanden
- In de psychiatrische instelling: …… dagen in de afgelopen 3 maanden
- In de andere instelling: …… dagen in de afgelopen 3 maanden

List of abbreviations

AE	Adverse Event(s)
AJCC	American Joint Committee on Cancer
ALC	Absolute Lymphocyte Count
ALT	Alanine-aminotransferase
AP	Alkaline phosphatase
AST	Aspartate-aminotransferase
Ca	Calcium
CCMO	Central Committee on Research Involving Human Subjects; in Dutch: Centrale Commissie Mensgebonden Onderzoek
CMV	Cytomegalovirus
CRP	C-reactive protein
CT	Computed Tomography
CTCAE	Common Terminology Criteria for Adverse Events
Creat	Creatinine
CRF	Case Report Form(s)
DC	Dendritic cell(s)
DTH	Delayed-type hypersensitivity
EBV	Epstein Barr virus
e-CRF	Electronic case report form(s)
EORTC	European organisation for research and treatment of cancer
EQ-5D	EuroQol-5D, a validated health-related quality of life instrument
Erasmus MC	Erasmus university medical centre Rotterdam
GCP	Good clinical practice
GGT	Υ-glutamyl transferase
GMP	Good manufacturing practice
Gp100	Glycoprotein 100
Hb	Hemoglobin
HBV	Hepatitis B virus
HCV	Hepatitis C virus

HIV	Human immunodeficiency virus
HLA	Human leukocyte antigen
HR	Hazard ratio
HRQoL	Health-related quality of life
Ht	Hematocrit
ICER	Incremental cost-effectiveness ratio(s)
ICH	International conference on harmonization of technical requirements for registration of pharmaceuticals for human use
ICMJE	International committee of medical journal editors
IFN	Interferon
IL	Interleukin
IMPD	Investigational Medicinal Product Dossier
ITT	Intention-to-treat
K	Potassium
KLH	Keyhole limpet hemocyanin
LDH	Lactate dehydrogenase
MAGE	Melanoma-associated antigen
MHC	Major histocompatibility complex
moDC	Monocyte-derived dendritic cell(s)
MRI	Magnetic resonance imaging
myDC	Myeloid dendritic cell(s)
nDC	Natural dendritic cell(s), i.e. plasmacytoid and/or myeloid dendritic cells
Na	Sodium
NKI	Netherlands cancer institute
NWO	Nederlandse organisatie voor wetenschappelijk onderzoek, the Netherlands organisation for scientific research
OS	Overall survival
P	Phosphatase
PBMC	Peripheral blood mononuclear cells
pDC	Plasmacytoid dendritic cell(s)
PET	Positron emission tomography

QLQ-C30	Quality of Life Questionnaire
QoL	Quality of life
QALY	Quality adjusted life year(s)
Radboudumc	Radboud university medical centre Nijmegen
RFS	Recurrence-free survival
RLND	Radical Lymph Node Dissection
SAE	Serious Adverse Event(s)
SUSAR	Suspected Unexpected Serious Adverse Reaction(s)
TDC	Trial coordination and data center
TLR	Toll-like receptor(s)
TPHA	Treponema pallidum hemagglutination assay, i.e. a serologic test for syphilis
ULN	Upper limit of laboratory normal range
VUmc	Free university medical centre Amsterdam
WBC	White blood cell(s)
WHO	World health orginazation
WMO	Medical Research Involving Human Subjects Act; in Dutch: Wet Medisch-wetenschappelijk Onderzoek met Mensen
WOCB	Women Of Child-Bearing potential

References

1. Edge, S.D.B., D.R. & Compton, C.C., *Melanoma of the skin*, in *AJCC Cancer Staging Manual*. 2010, Springer: New York.
2. Faries, M.B., et al., *Completion Dissection or Observation for Sentinel-Node Metastasis in Melanoma.* N Engl J Med, 2017. **376**(23): p. 2211-2222.
3. Balch, C.M., et al., *Final version of the American Joint Committee on Cancer staging system for cutaneous melanoma.* J Clin Oncol, 2001. **19**(16): p. 3635-48.
4. Verma, S., et al., *Systematic review of systemic adjuvant therapy for patients at high risk for recurrent melanoma.* Cancer, 2006. **106**(7): p. 1431-42.
5. Agarwala, S.S. and J.M. Kirkwood, *Adjuvant therapy of melanoma.* Semin Surg Oncol, 1998. **14**(4): p. 302-10.
6. Garbe, C., et al., *Systematic review of medical treatment in melanoma: current status and future prospects.* Oncologist, 2011. **16**(1): p. 5-24.
7. Eggermont, A.M., et al., *Adjuvant therapy with pegylated interferon alfa-2b versus observation alone in resected stage III melanoma: final results of EORTC 18991, a randomised phase III trial.* Lancet, 2008. **372**(9633): p. 117-26.
8. Wheatley, K., et al., *Does adjuvant interferon-alpha for high-risk melanoma provide a worthwhile benefit? A meta-analysis of the randomised trials.* Cancer Treat Rev, 2003. **29**(4): p. 241-52.
9. Eggermont, A.M., et al., *Adjuvant ipilimumab versus placebo after complete resection of high-risk stage III melanoma (EORTC 18071): a randomised, double-blind, phase 3 trial.* Lancet Oncol, 2015. **16**(5): p. 522-30.
10. Eggermont, A.M., et al., *Prolonged Survival in Stage III Melanoma with Ipilimumab Adjuvant Therapy.* N Engl J Med, 2016. **375**(19): p. 1845-1855.
11. Agrawal, S., et al., *The benefits of adjuvant radiation therapy after therapeutic lymphadenectomy for clinically advanced, high-risk, lymph node-metastatic melanoma.* Cancer, 2009. **115**(24): p. 5836-44.
12. Burmeister, B.H., et al., *Adjuvant radiotherapy versus observation alone for patients at risk of lymph-node field relapse after therapeutic lymphadenectomy for melanoma: a randomised trial.* Lancet Oncol, 2012. **13**(6): p. 589-97.
13. Kalialis, L.V., K.T. Drzewiecki, and H. Klyver, *Spontaneous regression of metastases from melanoma: review of the literature.* Melanoma Res, 2009. **19**(5): p. 275-82.
14. Penn, I., *Malignant melanoma in organ allograft recipients.* Transplantation, 1996. **61**(2): p. 274-8.
15. Azimi, F., et al., *Tumor-infiltrating lymphocyte grade is an independent predictor of sentinel lymph node status and survival in patients with cutaneous melanoma.* J Clin Oncol, 2012. **30**(21): p. 2678-83.
16. Banchereau, J. and R.M. Steinman, *Dendritic cells and the control of immunity.* Nature, 1998. **392**(6673): p. 245-52.
17. Figdor, C.G., et al., *Dendritic cell immunotherapy: mapping the way.* Nat Med, 2004. **10**(5): p. 475-80.
18. Tuyaerts, S., et al., *Current approaches in dendritic cell generation and future implications for cancer immunotherapy.* Cancer Immunol Immunother, 2007. **56**(10): p. 1513-37.
19. Lin, C.T., et al., *Maintenance of CD8 effector T cells by CD4 helper T cells eradicates growing tumors and promotes long-term tumor immunity.* Vaccine, 2006. **24**(37-39): p. 6199-207.

20. Knutson, K.L. and M.L. Disis, *Tumor antigen-specific T helper cells in cancer immunity and immunotherapy.* Cancer Immunol Immunother, 2005. **54**(8): p. 721-8.
21. Surman, D.R., et al., *Cutting edge: CD4+ T cell control of CD8+ T cell reactivity to a model tumor antigen.* J Immunol, 2000. **164**(2): p. 562-5.
22. Ossendorp, F., et al., *Specific T helper cell requirement for optimal induction of cytotoxic T lymphocytes against major histocompatibility complex class II negative tumors.* J Exp Med, 1998. **187**(5): p. 693-702.
23. Aarntzen, E.H., et al., *Skin-test infiltrating lymphocytes early predict clinical outcome of dendritic cell based vaccination in metastatic melanoma.* Cancer Res, 2012: p. 72:6102-10.
24. de Vries, I.J., et al., *Immunomonitoring tumor-specific T cells in delayed-type hypersensitivity skin biopsies after dendritic cell vaccination correlates with clinical outcome.* J Clin Oncol, 2005. **23**(24): p. 5779-87.
25. Breckpot, K., et al., *Dendritic cells differentiated in the presence of IFN-{beta} and IL-3 are potent inducers of an antigen-specific CD8+ T cell response.* J Leukoc Biol, 2005. **78**(4): p. 898-908.
26. Soruri, A., et al., *IL-4 down-regulates anaphylatoxin receptors in monocytes and dendritic cells and impairs anaphylatoxin-induced migration in vivo.* J Immunol, 2003. **170**(6): p. 3306-14.
27. MacDonald, K.P., et al., *Characterization of human blood dendritic cell subsets.* Blood, 2002. **100**(13): p. 4512-20.
28. Dzionek, A., et al., *BDCA-2, BDCA-3, and BDCA-4: three markers for distinct subsets of dendritic cells in human peripheral blood.* J Immunol, 2000. **165**(11): p. 6037-46.
29. Rissoan, M.C., et al., *Reciprocal control of T helper cell and dendritic cell differentiation.* Science, 1999. **283**(5405): p. 1183-6.
30. Cravens, P.D., et al., *Human peripheral blood dendritic cells and monocyte subsets display similar chemokine receptor expression profiles with differential migratory responses.* Scand J Immunol, 2007. **65**(6): p. 514-24.
31. Randolph, G.J., J. Ochando, and S. Partida-Sanchez, *Migration of dendritic cell subsets and their precursors.* Annu Rev Immunol, 2008. **26**: p. 293-316.
32. Yoneyama, H., et al., *Evidence for recruitment of plasmacytoid dendritic cell precursors to inflamed lymph nodes through high endothelial venules.* Int Immunol, 2004. **16**(7): p. 915-28.
33. Salio, M., et al., *Plasmacytoid dendritic cells prime IFN-gamma-secreting melanoma-specific CD8 lymphocytes and are found in primary melanoma lesions.* Eur J Immunol, 2003. **33**(4): p. 1052-62.
34. Fonteneau, J.F., et al., *Human immunodeficiency virus type 1 activates plasmacytoid dendritic cells and concomitantly induces the bystander maturation of myeloid dendritic cells.* J Virol, 2004. **78**(10): p. 5223-32.
35. Cantisani, R., et al., *Surface molecules on stimulated plasmacytoid dendritic cells are sufficient to cross-activate resting myeloid dendritic cells.* Hum Immunol, 2011. **72**(11): p. 1018-21.
36. Lou, Y., et al., *Plasmacytoid dendritic cells synergize with myeloid dendritic cells in the induction of antigen-specific antitumor immune responses.* J Immunol, 2007. **178**(3): p. 1534-41.
37. Steinman, R.M. and M. Pope, *Exploiting dendritic cells to improve vaccine efficacy.* J Clin Invest, 2002. **109**(12): p. 1519-26.
38. Mailliard, R.B., et al., *alpha-type-1 polarized dendritic cells: a novel immunization tool with optimized CTL-inducing activity.* Cancer Res, 2004. **64**(17): p. 5934-7.

39. Sporri, R. and C. Reis e Sousa, *Inflammatory mediators are insufficient for full dendritic cell activation and promote expansion of CD4+ T cell populations lacking helper function.* Nat Immunol, 2005. **6**(2): p. 163-70.
40. Goriely, S., M.F. Neurath, and M. Goldman, *How microorganisms tip the balance between interleukin-12 family members.* Nat Rev Immunol, 2008. **8**(1): p. 81-6.
41. Ito, T., et al., *Interferon-alpha and interleukin-12 are induced differentially by Toll-like receptor 7 ligands in human blood dendritic cell subsets.* J Exp Med, 2002. **195**(11): p. 1507-12.
42. Aarntzen, E.H., et al., *Targeting CD4(+) T-helper cells improves the induction of antitumor responses in dendritic cell-based vaccination.* Cancer Res, 2013. **73**(1): p. 19-29.
43. Banchereau, J., et al., *Immune and clinical outcomes in patients with stage IV melanoma vaccinated with peptide-pulsed dendritic cells derived from CD34+ progenitors and activated with type I interferon.* J Immunother, 2005. **28**(5): p. 505-16.
44. Nestle, F.O., et al., *Vaccination of melanoma patients with peptide- or tumor lysate-pulsed dendritic cells.* Nat Med, 1998. **4**(3): p. 328-32.
45. Thurner, B., et al., *Vaccination with mage-3A1 peptide-pulsed mature, monocyte-derived dendritic cells expands specific cytotoxic T cells and induces regression of some metastases in advanced stage IV melanoma.* J Exp Med, 1999. **190**(11): p. 1669-78.
46. Markowicz, S., et al., *Adjuvant vaccination with melanoma antigen-pulsed dendritic cells in stage III melanoma patients.* Med Oncol, 2012. **29**(4): p. 2966-77.
47. De Vries, I.J., et al., *Effective migration of antigen-pulsed dendritic cells to lymph nodes in melanoma patients is determined by their maturation state.* Cancer Res, 2003. **63**(1): p. 12-7.
48. de Vries, I.J., et al., *Maturation of dendritic cells is a prerequisite for inducing immune responses in advanced melanoma patients.* Clin Cancer Res, 2003. **9**(14): p. 5091-100.
49. Bol, K.F., et al., *Favorable overall survival in stage III melanoma patients after adjuvant dendritic cell vaccination.* OncoImmunology, 2015.
50. Tel, J., et al., *Natural human plasmacytoid dendritic cells induce antigen-specific T-cell responses in melanoma patients.* Cancer Res, 2013. **73**(3): p. 1063-75.
51. Schadendorf, D., et al., *Dacarbazine (DTIC) versus vaccination with autologous peptide-pulsed dendritic cells (DC) in first-line treatment of patients with metastatic melanoma: a randomized phase III trial of the DC study group of the DeCOG.* Ann Oncol, 2006. **17**(4): p. 563-70.
52. Balch, C.M., et al., *Final version of 2009 AJCC melanoma staging and classification.* J Clin Oncol, 2009. **27**(36): p. 6199-206.
53. Cornish, D., et al., *A systematic review of health-related quality of life in cutaneous melanoma.* Ann Oncol, 2009. **20 Suppl 6**: p. vi51-8.
54. Aaronson, N.K., et al., *The European Organization for Research and Treatment of Cancer QLQ-C30: a quality-of-life instrument for use in international clinical trials in oncology.* J Natl Cancer Inst, 1993. **85**(5): p. 365-76.
55. Osoba, D., et al., *Interpreting the significance of changes in health-related quality-of-life scores.* J Clin Oncol, 1998. **16**(1): p. 139-44.
56. Guy, G.P., Jr., et al., *Melanoma treatment costs: a systematic review of the literature, 1990-2011.* Am J Prev Med, 2012. **43**(5): p. 537-45.
