# Supplementary material for: Adjuvant dendritic cell therapy in stage IIIB/C melanoma: the MIND-DC randomized phase III trial
Source: Nat Commun. 2024 Feb 23;15:1632. doi: 10.1038/s41467-024-45358-0 (PMC10891118; doi:10.1038/s41467-024-45358-0)
Supplement: Supplementary file 3 — Reporting Summary [file 41467_2024_45358_MOESM3_ESM.pdf]

nature portfolio

Corresponding author(s): Jolanda de Vries

Last updated by author(s): Jul 9, 2023

Reporting Summary

Nature Portfolio wishes to improve the reproducibility of the work that we publish. This form provides structure for consistency and transparency in reporting. For further information on Nature Portfolio policies, see our Editorial Policies and the Editorial Policy Checklist.

Statistics

For all statistical analyses, confirm that the following items are present in the figure legend, table legend, main text, or Methods section.

n/a	Confirmed	
☐	☒	The exact sample size (*n*) for each experimental group/condition, given as a discrete number and unit of measurement
☒	☐	A statement on whether measurements were taken from distinct samples or whether the same sample was measured repeatedly
☐	☒	The statistical test(s) used AND whether they are one- or two-sided<br>*Only common tests should be described solely by name; describe more complex techniques in the Methods section.*
☒	☐	A description of all covariates tested
☒	☐	A description of any assumptions or corrections, such as tests of normality and adjustment for multiple comparisons
☐	☒	A full description of the statistical parameters including central tendency (e.g. means) or other basic estimates (e.g. regression coefficient) AND variation (e.g. standard deviation) or associated estimates of uncertainty (e.g. confidence intervals)
☐	☒	For null hypothesis testing, the test statistic (e.g. *F*, *t*, *r*) with confidence intervals, effect sizes, degrees of freedom and *P* value noted<br>*Give P values as exact values whenever suitable.*
☒	☐	For Bayesian analysis, information on the choice of priors and Markov chain Monte Carlo settings
☒	☐	For hierarchical and complex designs, identification of the appropriate level for tests and full reporting of outcomes
☒	☐	Estimates of effect sizes (e.g. Cohen's *d*, Pearson's *r*), indicating how they were calculated

Our web collection on statistics for biologists contains articles on many of the points above.

Software and code

Policy information about availability of computer code

Data collection	Trial data was collected and managed using the CASTOR electronic data capture platform (castoredc.com), and exported from CASTOR to CSV files for further processing.
Data analysis	All statistical analysis was performed using the R platform for statistical computing, version 4.2. Survival data were analyzed using the R package "survival" which is included in the R platform, and visualized using the R packages "survminer", version 0.4.9., and "ggplot2", version 3.1.1.

For manuscripts utilizing custom algorithms or software that are central to the research but not yet described in published literature, software must be made available to editors and reviewers. We strongly encourage code deposition in a community repository (e.g. GitHub). See the Nature Portfolio guidelines for submitting code & software for further information.

Data

Policy information about availability of data

All manuscripts must include a data availability statement. This statement should provide the following information, where applicable:

- Accession codes, unique identifiers, or web links for publicly available datasets
- A description of any restrictions on data availability
- For clinical datasets or third party data, please ensure that the statement adheres to our policy

Source data for all figures (i.e., the numbers being tabulated or graphically displayed) will be made available as Supplementary information upon acceptance of this paper. Individual participant data are not made publicly available owing to privacy and ethical restrictions. Specific requests for access to raw and/or analyzed data

should be sent to the corresponding author. Data requests will be reviewed by the principal investigators of the trial. Any data and materials that can be shared will require approval from the Institutional Review Board and a data or material transfer agreement.

Research involving human participants, their data, or biological material

Policy information about studies with human participants or human data. See also policy information about sex, gender (identity/presentation), and sexual orientation and race, ethnicity and racism.

Reporting on sex and gender	Sex and gender were not considered in the study design and analyses.
Reporting on race, ethnicity, or other socially relevant groupings	Not reported.
Population characteristics	Relevant patient characteristics are described in Table 1, including tumor stage, performance status, BRAF status, HLA status and previous cancer treatment.
Recruitment	Study was open to all patients with stage III melanoma in the Netherlands and could be reffered from all centers in the Netherlands. No selection was made.
Ethics oversight	Dutch Central Committee on Research Involving Human Subjects approved the protocol.

Note that full information on the approval of the study protocol must also be provided in the manuscript.

Field-specific reporting

Please select the one below that is the best fit for your research. If you are not sure, read the appropriate sections before making your selection.

☒ Life sciences ☐ Behavioural & social sciences ☐ Ecological, evolutionary & environmental sciences

For a reference copy of the document with all sections, see nature.com/documents/nr-reporting-summary-flat.pdf

Life sciences study design

All studies must disclose on these points even when the disclosure is negative.

Sample size	To detect an improvement in the 2-year RFS rate from an estimated 50% to 70%, with a power of 80% and two-sided α level of 0.05, we planned to randomly assign 210 patients.
Data exclusions	non-eligible patients were excluded from the analyses as predefined in the protocol.
Replication	Not applicable: Survival data is not replicable.
Randomization	Patients were randomly assigned (2:1) to receive nDC therapy or placebo. Central randomisation was based on a minimisation technique as described by Pockock et al. Patients were stratified by disease stage (IIIB vs IIIC), adjuvant radiotherapy (yes vs no), BRAFV600 mutation status (BRAF mutant vs BRAF wildtype), and HLA-type (HLA-A02:01 negative vs HLA-A02:01 positive).
Blinding	Patients treatment allocation was masked for patients and clinical investigators. Only laboratory personnel, pharmacists, and statisticians were aware of group assignment.

Reporting for specific materials, systems and methods

We require information from authors about some types of materials, experimental systems and methods used in many studies. Here, indicate whether each material, system or method listed is relevant to your study. If you are not sure if a list item applies to your research, read the appropriate section before selecting a response.

Materials & experimental systems

n/a	Involved in the study	
☐	☒	Antibodies
☒	☐	Eukaryotic cell lines
☒	☐	Palaeontology and archaeology
☒	☐	Animals and other organisms
☐	☒	Clinical data
☒	☐	Dual use research of concern
☒	☐	Plants

Methods

n/a	Involved in the study	
☒	☐	ChIP-seq
☒	☐	Flow cytometry
☒	☐	MRI-based neuroimaging

Antibodies

Antibodies used	A list of antibodies is added as a separate document "antibodies MIND-DC"
Validation	The link with all the validation information for each antibody is incorporated in the document "antibodies MIND-DC"

Clinical data

Policy information about clinical studies

All manuscripts should comply with the ICMJEguidelines for publication of clinical research and a completedCONSORT checklist must be included with all submissions.

Clinical trial registration	This trial is registered with EudraCT, number 2015-002531-29, and ClinicalTrials.gov, number NCT02993315
Study protocol	Accessible (submitted with manuscript).
Data collection	PThe MIND-DC study is a double-blind, randomised, placebo-controlled phase 3 study performed in two centers in the Netherlands (Radboud university medical center, Nijmegen and Isala, Zwolle).<br>Patient recruiment: Between December 2016 and November 2018.<br>Data collection: ongoing (5 years follow-up).
Outcomes	The primary endpoint was the 2-year RFS rate, defined as the percentage of patients who are alive and without recurrence of disease two years after randomization, compared to treatment with matching placebo. Patients were planned to be assessed for recurrence of disease every 3 months during the first 2 years and every 6 months thereafter up to 5 years. Disease assessment consisted of physical examination and CT scans. Other imaging techniques were used as clinically indicated. Recurrent disease was histologically confirmed, whenever possible.<br>Secondary endpoints were median RFS, 2-year and median OS, adverse event (AE) profile, and immunological response. Adverse events were recorded using the Common Toxicity Criteria for Adverse Events version 4.03 up to 30 days after the last administration of study treatment or start of another cancer therapy, whichever occurred first. Serious adverse events believed to be related to the study treatment were still recorded after this period. Apheresis-related AEs are defined as all related AEs within one week of apheresis. AEs are considered related to the treatment/apheresis if the event was recorded as possible, probable, or definite related to the apheresis procedure by the treating physician.<br>The Kaplan-Meier method was used to estimate median RFS and OS distributions and the 90% CI of these estimates. A comparison between the groups was made using the log-rank test. Hazard ratios were estimated with a Cox proportional hazards model, stratified by stage of the disease, adjuvant radiotherapy, BRAF mutation status, and HLA-type. RFS was defined as the time between randomization and the date of first recurrence (local, regional, or distant metastasis) or death, whichever occurred first. P values for differences between fractions (such as the fraction of patients showing an immune response in the nDC treatment group versus the control group) were calculated by means of the chi-square test. When events had not occurred, survival was censored at the date of last follow-up. We calculated median follow-up using the inverse Kaplan-Meier method. Efficacy analysis was performed on the intention-to-treat population, defined as all eligible patients assessed in the group they were allocated by randomisation. The safety population consisted of all patients who at least started apheresis.
